# Supplementary material for: Functional divergence of a bacterial enzyme promotes healthy or acneic skin
Source: Nat Commun. 2023 Dec 5;14:8061. doi: 10.1038/s41467-023-43833-8 (PMC10697930; doi:10.1038/s41467-023-43833-8)
Supplement: Supplementary file 1 — Supplementary Information [file 41467_2023_43833_MOESM1_ESM.pdf]

Functional divergence of a bacterial enzyme promotes healthy or acneic skin

Irshad A. Hajam[1,#], Madhusudhanarao Katiki[2,#], Randall McNally[2,6,#], María Lázaro-Díez[1,7,#], Stacey Kolar[2,8,#], Avradip Chatterjee[2], Cesia Gonzalez[1], Mousumi Paulchakrabarti[3], Biswa Choudhury[3], JR Caldera[1,2,9], Desmond Trieu[1,10], Chih-Ming Tsai[1], Xin Du[1], Huiying Li[4], Ramachandran Murali[2], George Y. Liu[1,5]

[1]Department of Pediatrics, University of California San Diego, San Diego, CA 92093, USA

[2]Department of Biomedical Sciences, Research Division of Immunology, Cedars-Sinai Medical Center, Los Angeles, CA 90048, USA

[3]GlycoAnalytics Core, University of California San Diego, San Diego, CA 92093, USA

[4]Department of Molecular and Medical Pharmacology, Crump Institute for Molecular Imaging, David Geffen School of Medicine, UCLA, Los Angeles, CA 90095, USA

[5]Division of Infectious Diseases, Rady Children's Hospital, San Diego, CA 92123, USA

[6]Vault Pharma Inc. 570 Westwood Plaza, Los Angeles, CA 90025, USA

[7]AIDS Research Institute (IrsiCaixa). VIRus Immune Escape and VACcine Design (VIRIEVAC) Universitary Hospital German Trias i Pujol Crta Canyet s/n 08916, Badalona, Barcelona, Spain

[8]Pharmacology at Armata Pharmaceuticals, Inc. Marina del Rey, CA 90292, USA

[9]Department of Pathology & Laboratory Medicine, UCLA Health & David Geffen School of Medicine, Los Angeles, CA 90095, USA

[10]School of Pharmacy, University of California San Francisco, San Francisco, CA 94143,USA

[#]These authors contributed equally.

Correspondence:

George Y. Liu (gyliu@health.ucsd.edu); Ramachandran Murali (ramachandran.murali@csmc.edu)

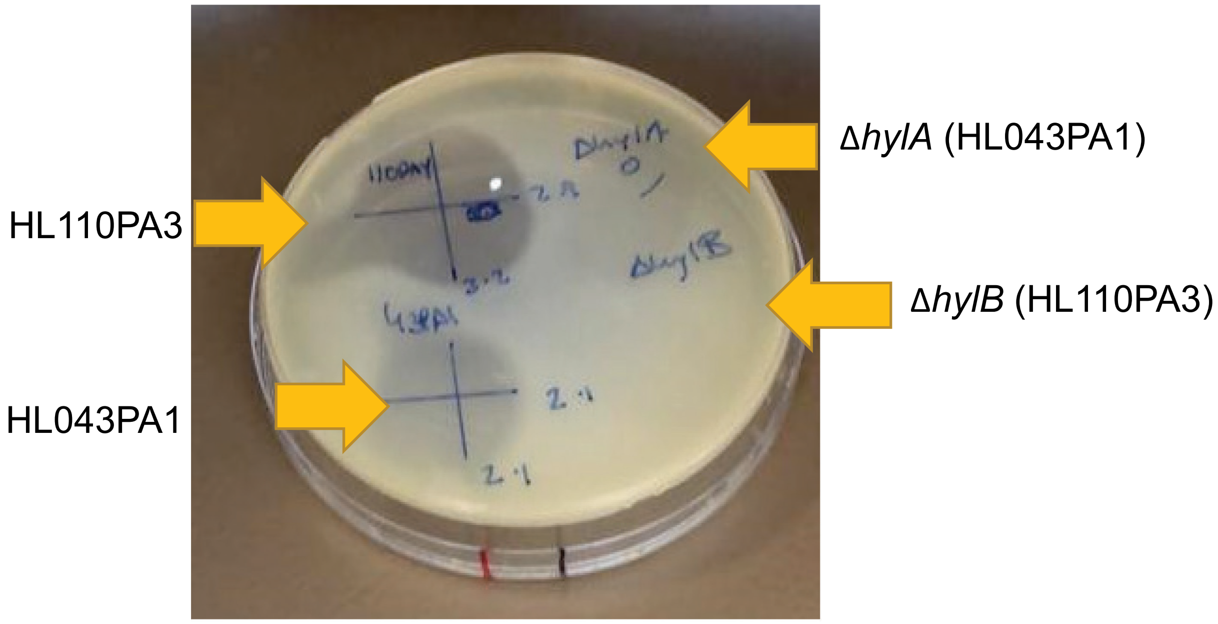

Supplementary Fig. 1: Verification of Δ*HylA* and Δ*HylB* enzymatic activity. Culture supernatants from wild-type or isogenic mutants were tested for HA degrading activity on an HA agar plate. Arrows show areas of HA clearance from incubation with WT strains, absent on plates with Δ*Hyl* strains. The results are representative of at least two independent experiments.

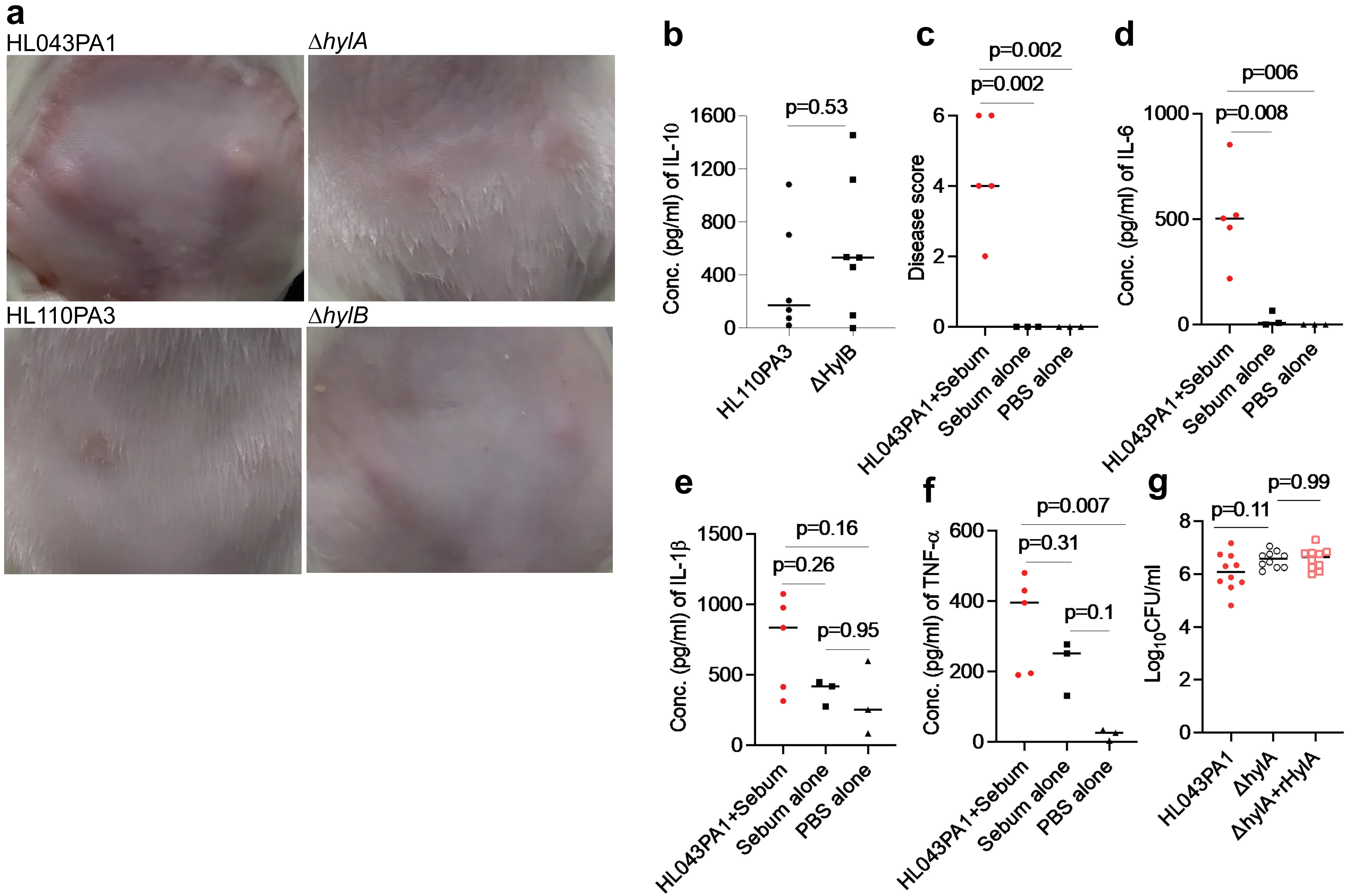

Supplementary Fig. 2: HylA enzyme is a major virulence factor in acne pathogenesis. a-b, CD1 mice were infected i.d. with WT (HL043PA1 or HL110PA3) or isogenic mutant (Δ*hylA* or Δ*hylB*) *C. acnes* ($2x10^7$cfu/mouse). **(a)** Representative images of skin lesions at 2 d post-infection. **(b)** IL-10 level (n=6 for HL110PA3 and n=7 for ΔHylB). **c-e,** CD1 mice (n=3 for sebum and PBS, and n=5 for HL043PA1 + sebum) were infected i.d. with HL043P1 plus topically applied sebum or topically applied sebum only or no treatment, and then disease score **(c)** and cytokines **(d-f)** were measured on d2 (48 hr). **g,** CD1 mice (n=10) were infected as above with HL043PA1, Δ*hylA* or Δ*hylA* plus rHylA protein (10 µg), and CFU from skin lesions at 2d post-infection was analyzed. Bars denote median and data were from one to two independent experiments. Data were analyzed by non-parametric two-tailed Mann-Whitney U test **(b)** or by one-way ANOVA with Tukey`s post-hoc test **(c-g)**.

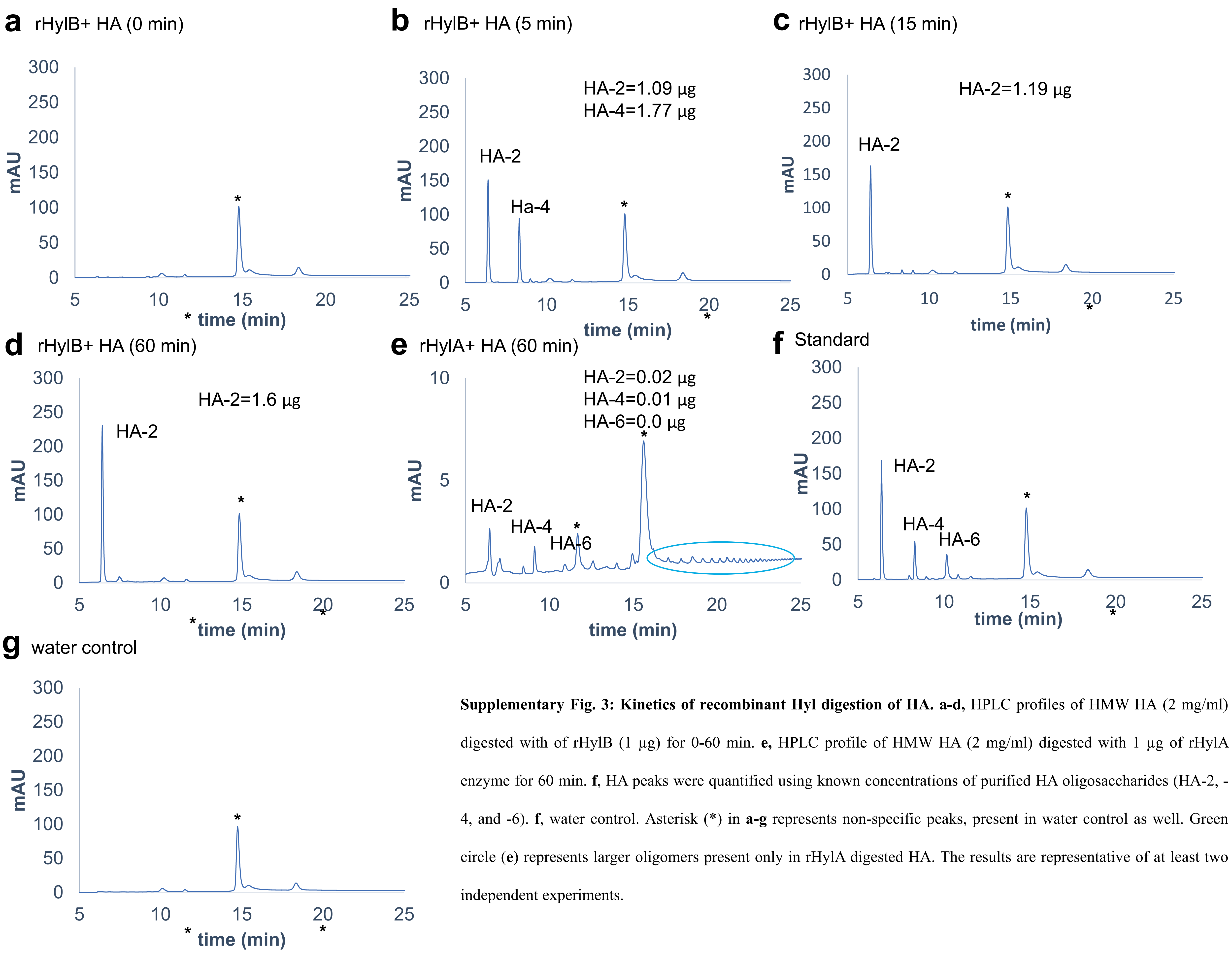

Supplementary Fig. 3: Kinetics of recombinant Hyl digestion of HA. a-d, HPLC profiles of HMW HA (2 mg/ml) digested with of rHylB (1 µg) for 0-60 min. **e,** HPLC profile of HMW HA (2 mg/ml) digested with 1 µg of rHylA enzyme for 60 min. **f**, HA peaks were quantified using known concentrations of purified HA oligosaccharides (HA-2, -4, and -6). **f**, water control. Asterisk (*) in **a-g** represents non-specific peaks, present in water control as well. Green circle (**e**) represents larger oligomers present only in rHylA digested HA. The results are representative of at least two independent experiments.

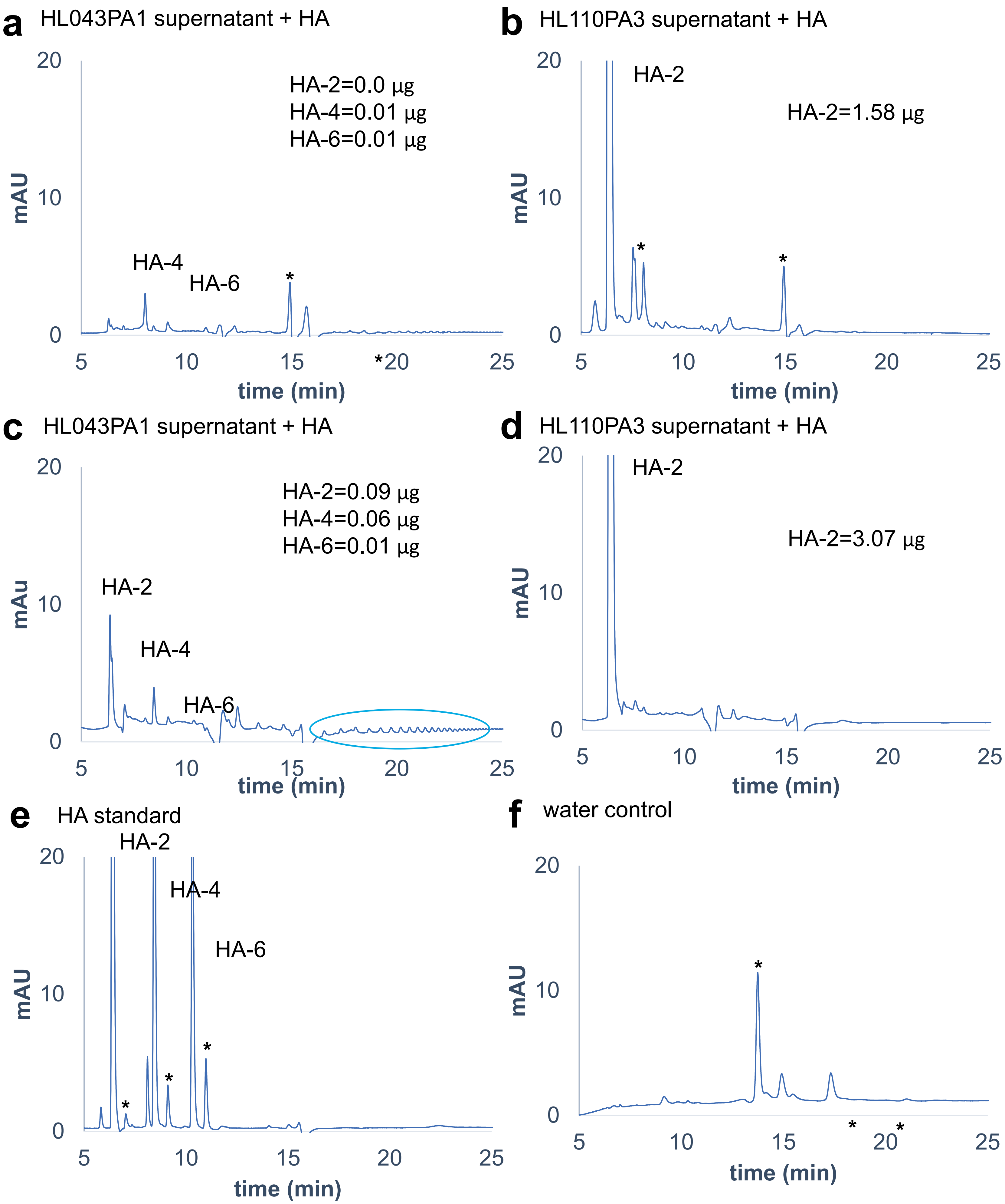

Supplementary Fig. 4: Kinetics of HA digestion with supernatant from either HylA or HylB expressing *C. acnes* strains. a-d, HMW HA substrate (2 mg/ml) was digested with supernatant (10 µl) from either HL043PA1 or HL110PA3 for 1 hr (**a, b**) or 24 hr (**c, d**), followed by HPLC analysis. **e**, digested HA peaks (HA-2, -4 and -6) were quantified using known concentrations of purified HA oligosaccharides. **f,** water control. Asterisk (*) in **a-f** represents non-specific peaks, present in water control as well. Green circle (**b**) represents larger oligomers present only in HA digested with the HL043PA1 supernatant at 24 hr. The results are representative of at least two independent experiments.

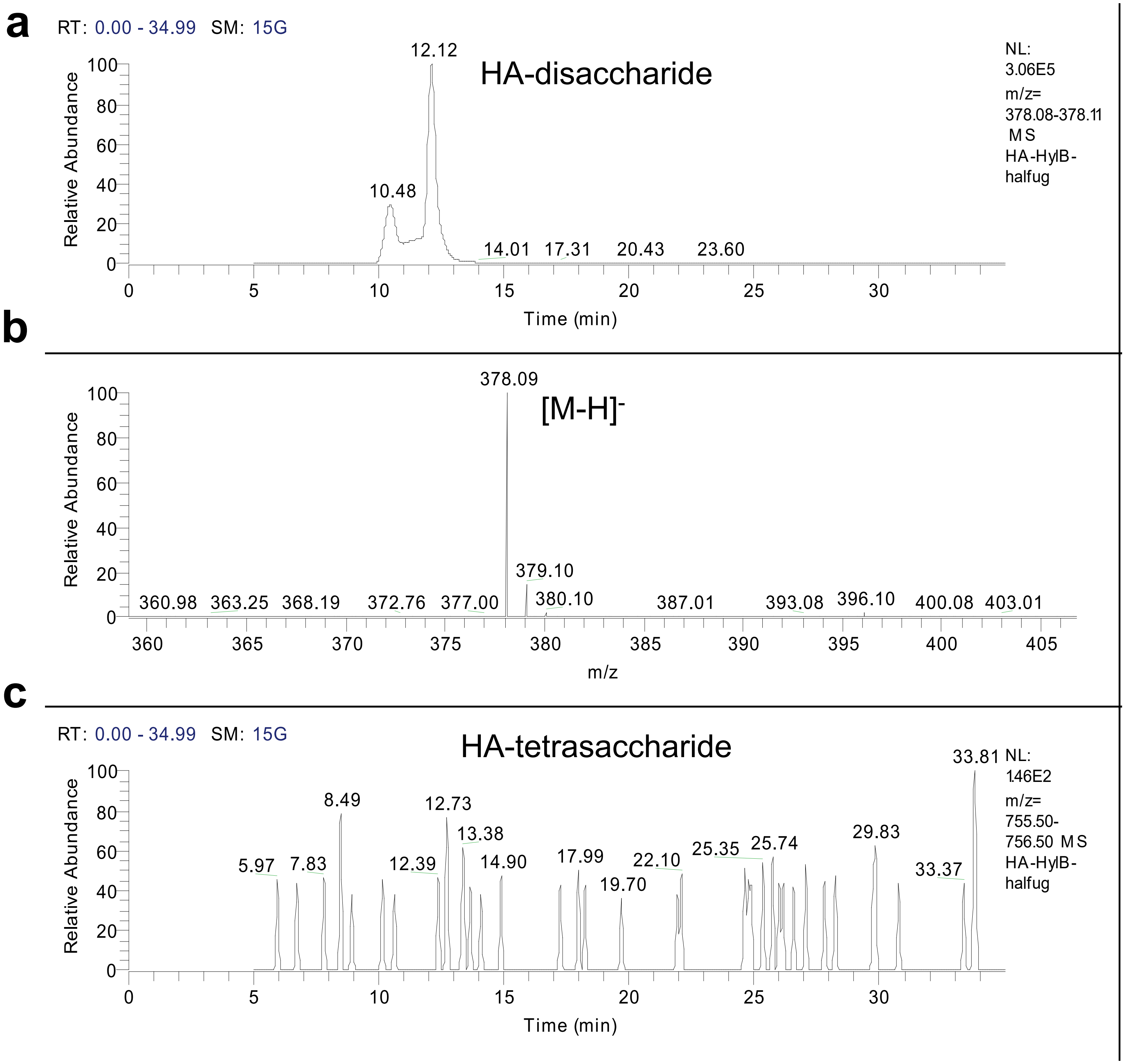

Supplementary Fig. 5: HylB digest analyzed by HPLC and LC-MS demonstrates only HA-disaccharide. a, HPLC of HylB digest (50ug HA+1ug HylB for 24 hr) demonstrates 2 peaks, 12.12 and 10.48, which correspond to the isomeric forms of HA-disaccharide. **b,** LC-MS showing that the peak from (a) is HA-disaccharide by m/z. **c,** HPLC shows no evidence of HA-tetra-saccharide from HylB digest.

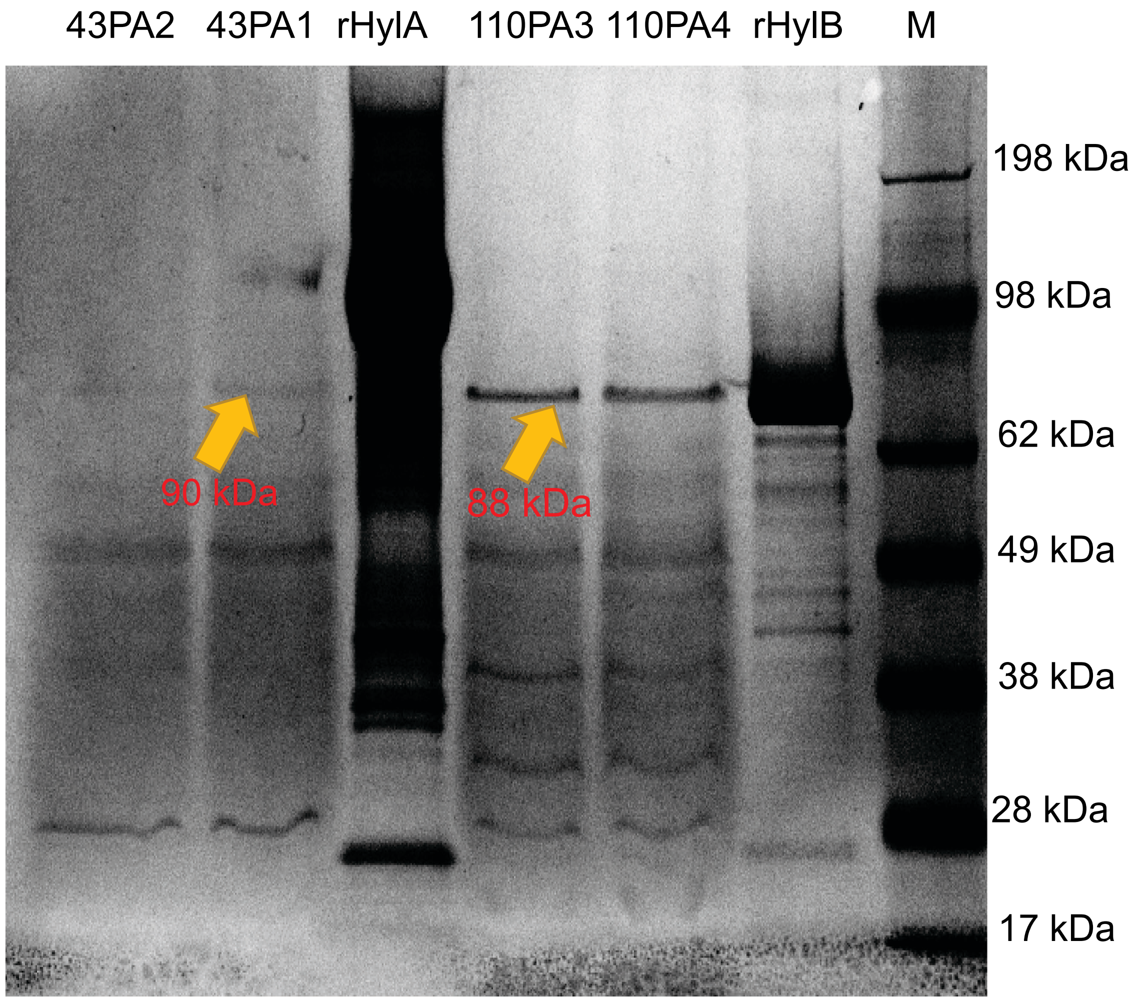

Supplementary Fig. 6: HylA and HylB protein level in health- and acne- associated *C. acnes* strains. Two acne- (HL043PA1 and HL043PA2) and two health- (HL110PA3 and HL110PA4) associated strains were grown for 4 days, and supernatants were analyzed by SDS-PAGE for Hyl expression with rHylA and rHylB proteins as positive controls. The results are representative of at least two independent experiments. The yellow arrows indicate expressed HylA and HylB. The photo is contrast-enhanced equally for both HylA and HylB proteins.

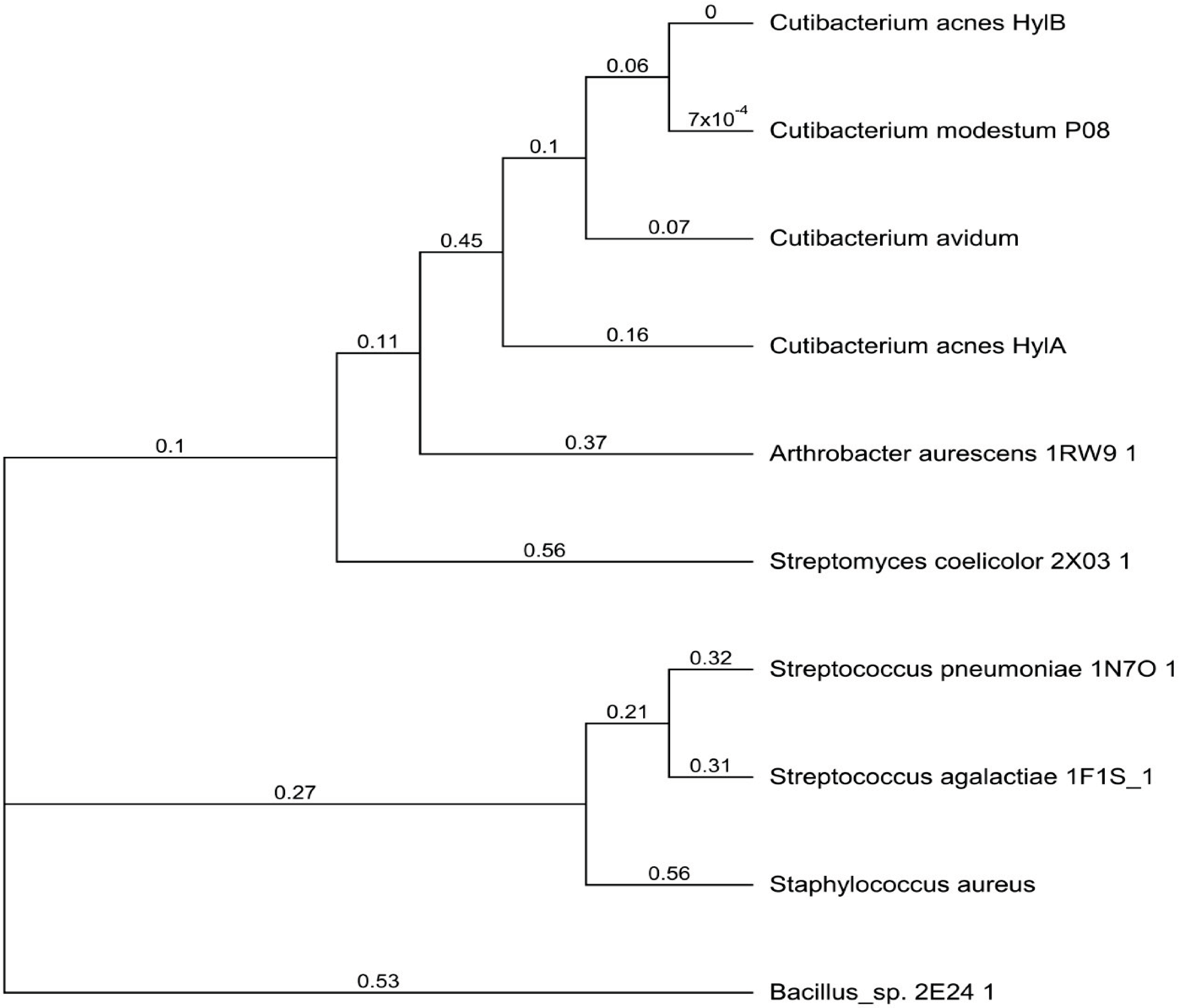

Supplementary Fig. 7: Hyl phylogenetic tree. Phylogenetic tree built using Geneious Prime.

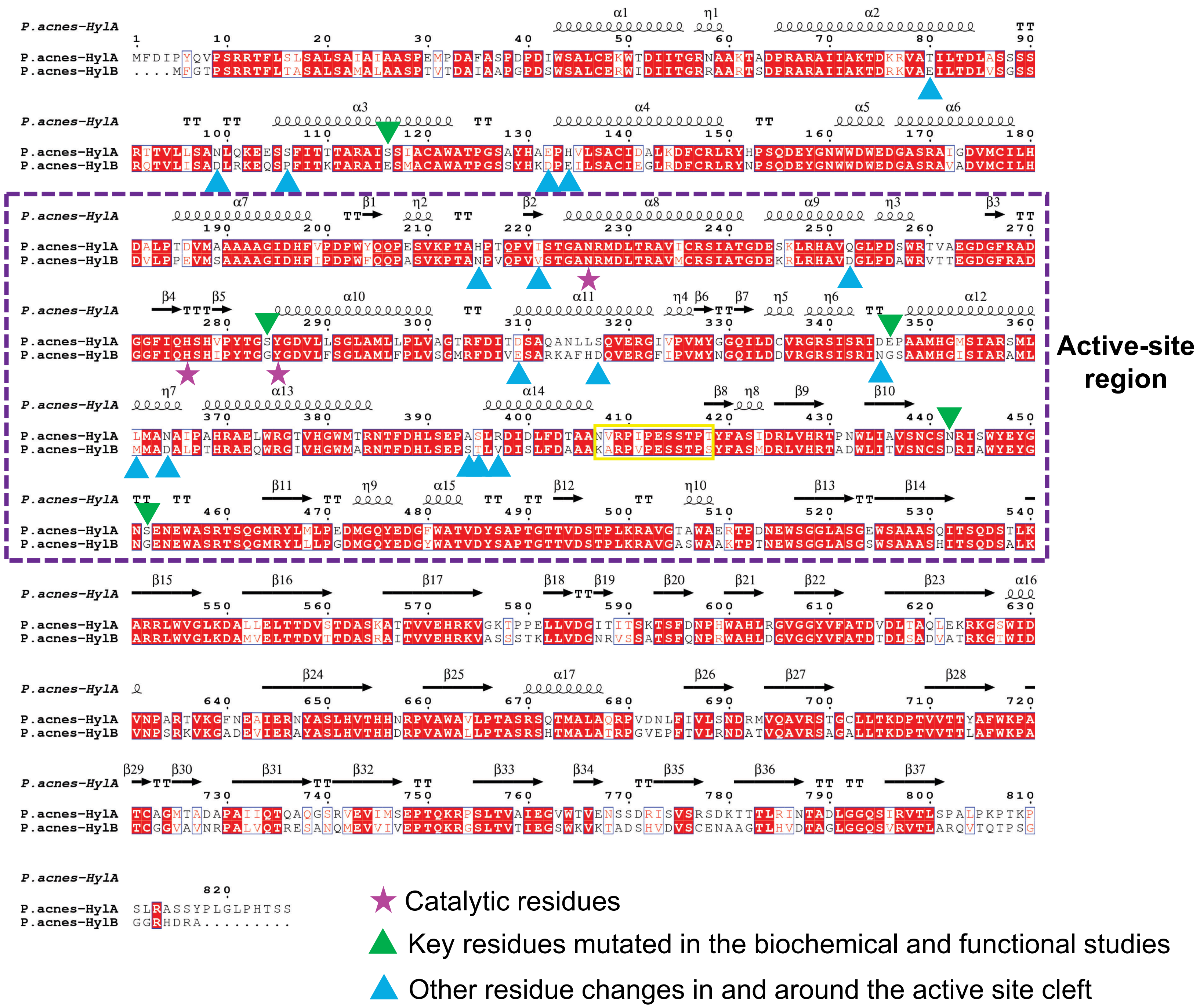

Supplementary Fig. 8: Multiple sequence alignment of HylA and HylB. The amino acid sequences of HylA and HylB are compared through multiple sequence alignment by using Clustal Omega server [https://www.ebi.ac.uk/Tools/msa/clustalo/]. The active site region is highlighted by a dashed purple rectangle and the inter-domain linker 407N-418T (HylA numbering) is shown by a gray rectangle. The catalytic residues 226N, 276H, and 285Y (HylA numbering) are marked by magenta asterisk. The significant amino acid differences between HylA and HylB, that are mutated in the biochemical and functional studies are marked by green triangles, while the other residue changes are marked by light blue triangles. Completely conserved residues are shown in red boxes with white characters, while the residues in the white boxes with red characters are relatively conserved. Secondary structural elements of HylA are labeled above the sequences.

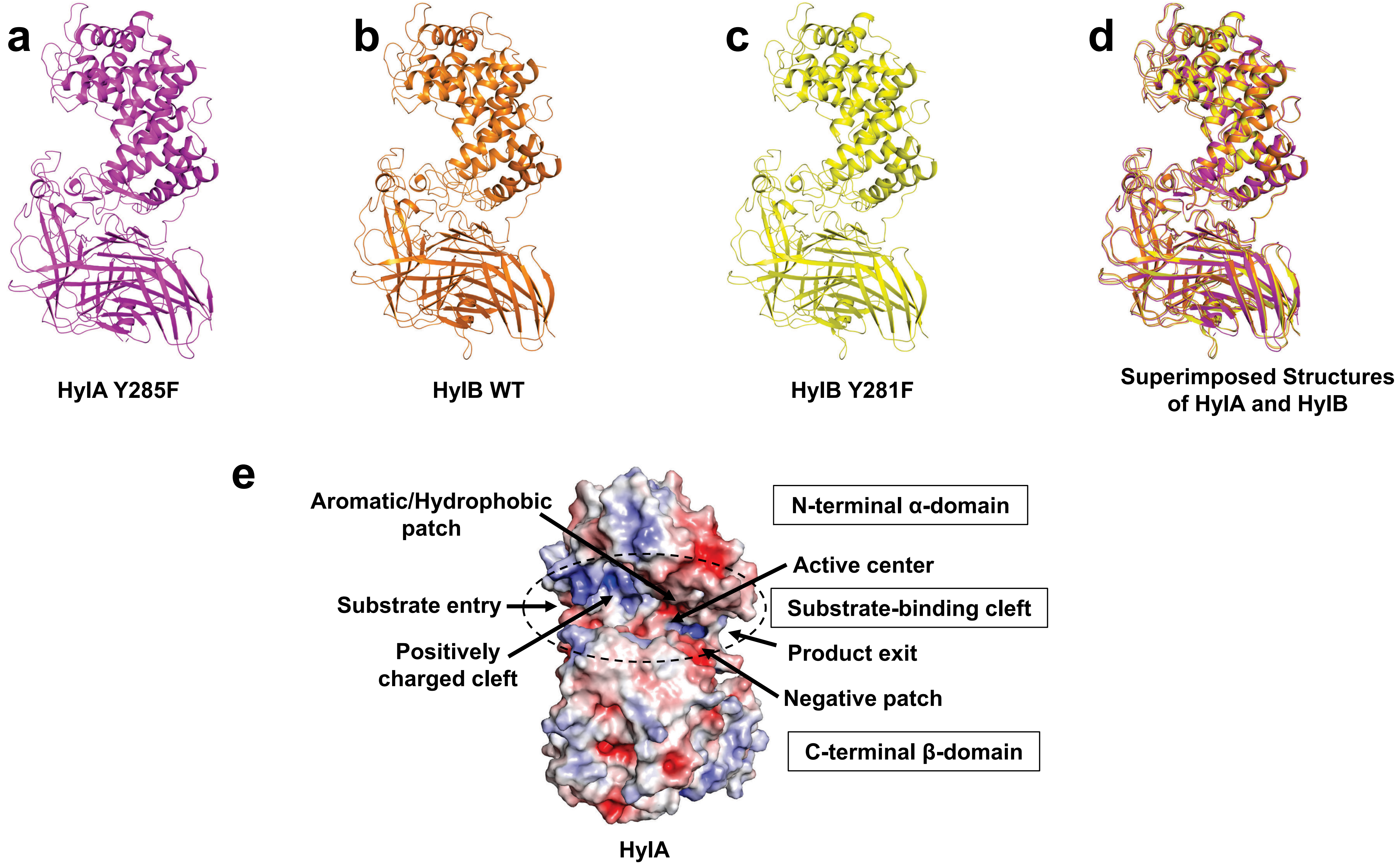

Supplementary Fig. 9: Crystal structures of HylA and HylB, and functional regions of Hyl from *C. acnes*. a-c, HylA and HylB (wild-type and mutant) crystal structures are shown. **d,** superimposition of the three crystal structures of HylA (PDB: 8FYG[https://www.rcsb.org/structure/unreleased/8FYG]) and HylB (wild-type (PDB: 8FNX[https://www.rcsb.org/structure/unreleased/8FNX]) and mutant (PDB: 8G0O[https://www.rcsb.org/structure/unreleased/8G0O])) crystal structures are shown. **d,** superimposition of the three crystal structures of HylA and HylB are shown superimposed. **e,** the putative functional parts of the Hyl enzyme from *C. acnes* are shown in the electrostatic surface view (prepared by APBS Electrostatics, PyMOL Molecular Graphics System, Version 2.4 Schrödinger, LLC.) of the HylA. All these regions, including positively charged cleft area, aromatic/hydrophobic patch, active center, and negative patch, are located around the substrate-binding cleft. It also depicts the substrate entry and products release ports at the cleft region. These structural regions in the homologous enzymes, including ScHyl (PDB: 2X03) [17], SpnHyl (PDB: 2BRW) [20], and SaHyl (PDB: 1F1S) [21], were shown to be involved in substrate attraction, binding, positioning and translocation, and product release.

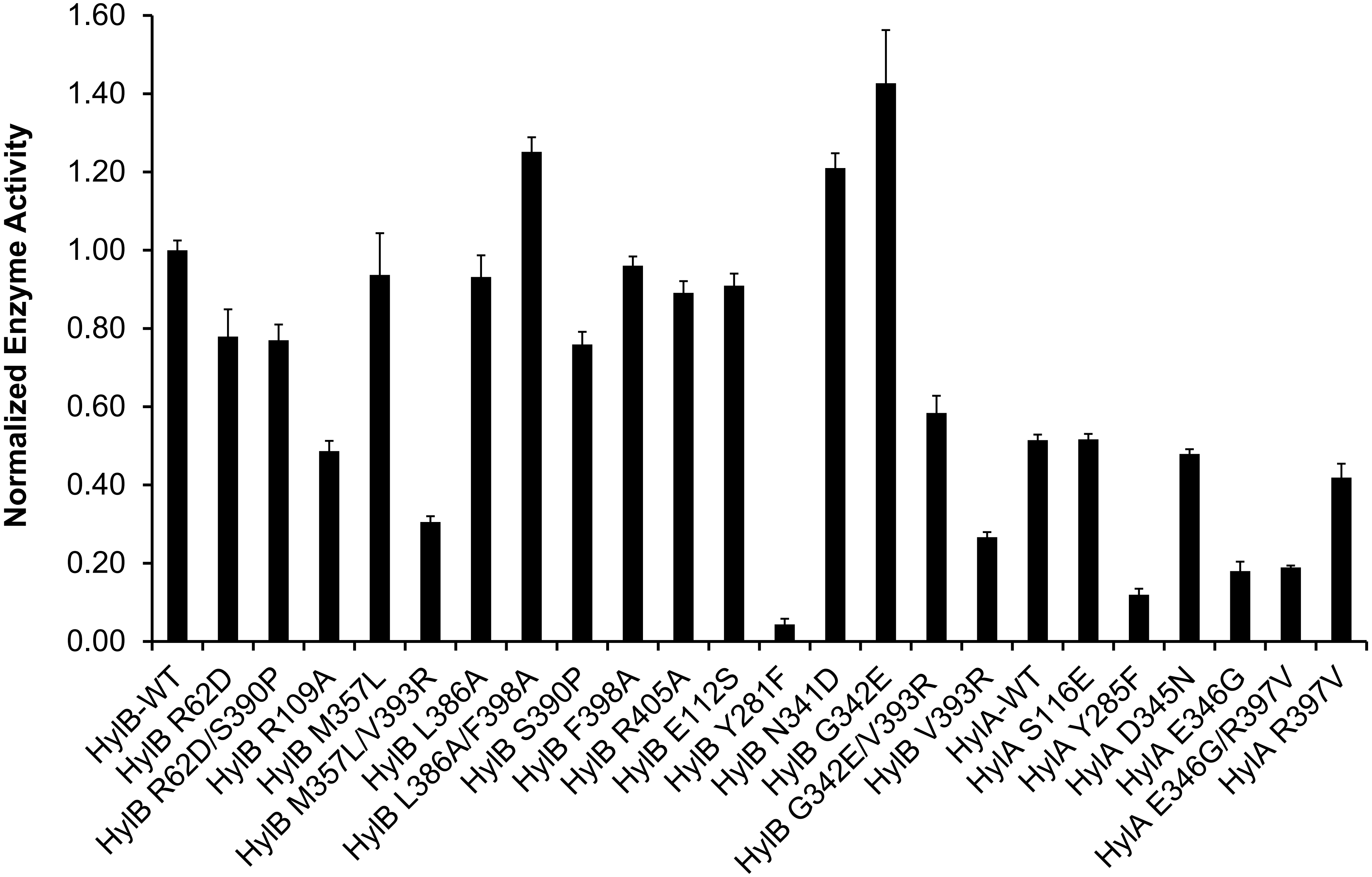

Supplementary Fig. 10: Enzyme activity of HylA and HylB with mutations. HylB at concentration 15 ng/mL and high molecular weight hyaluronic acid at concentration 0.2 mg/mL were monitored at wavelength 232 nm. Reaction volume was 100 µL. Assay buffer contained 100 mM Na acetate pH 5.5, 10 mM $CaCl_2$, and 0.5 mM TCEP. Results are scaled relative to wild-type HylB. Error bars are standard error of three measurements.

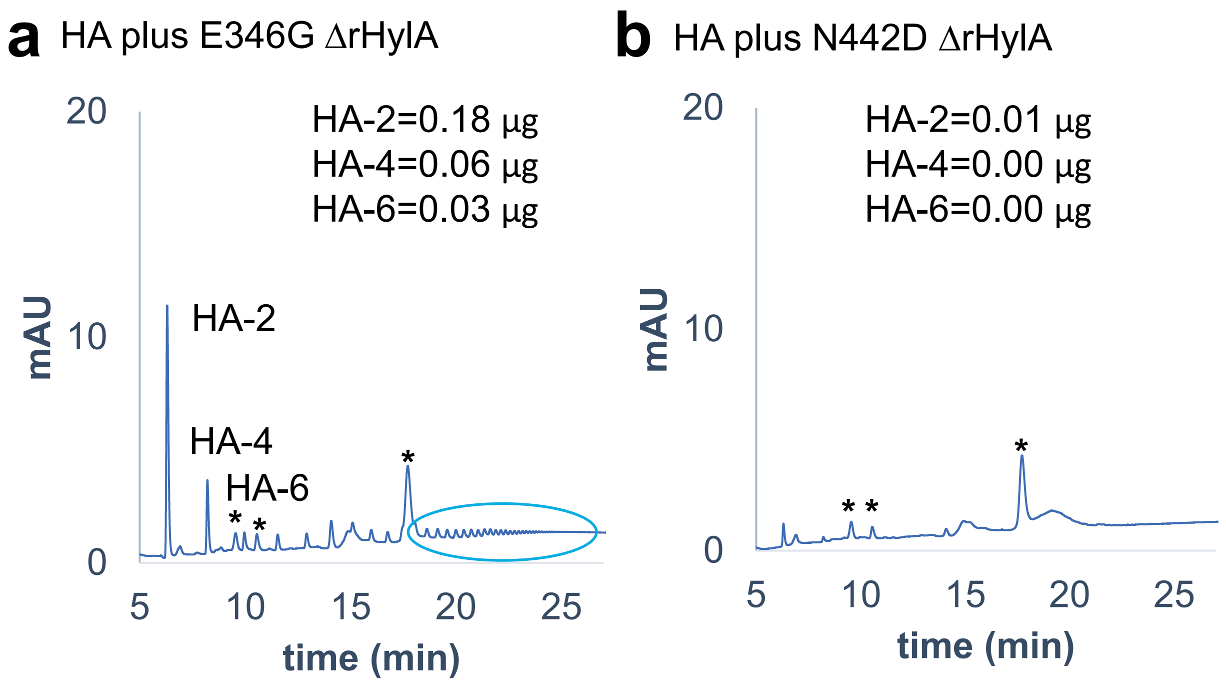

Supplementary Fig. 11: HA degradation product from HA incubation with mutant rHylA proteins. a-b, HPLC profiles of HMW HA (2 mg/ml plus 0.35 µg recombinant protein) digested for 24 hr with mutant N442D rHylA (a) or E346G (**b**). Asterisk (*) in **a, b** represents non-specific peaks, present in water control as well. Green circle (**a**) shows larger oligomers. Data are representative of two independent experiments.

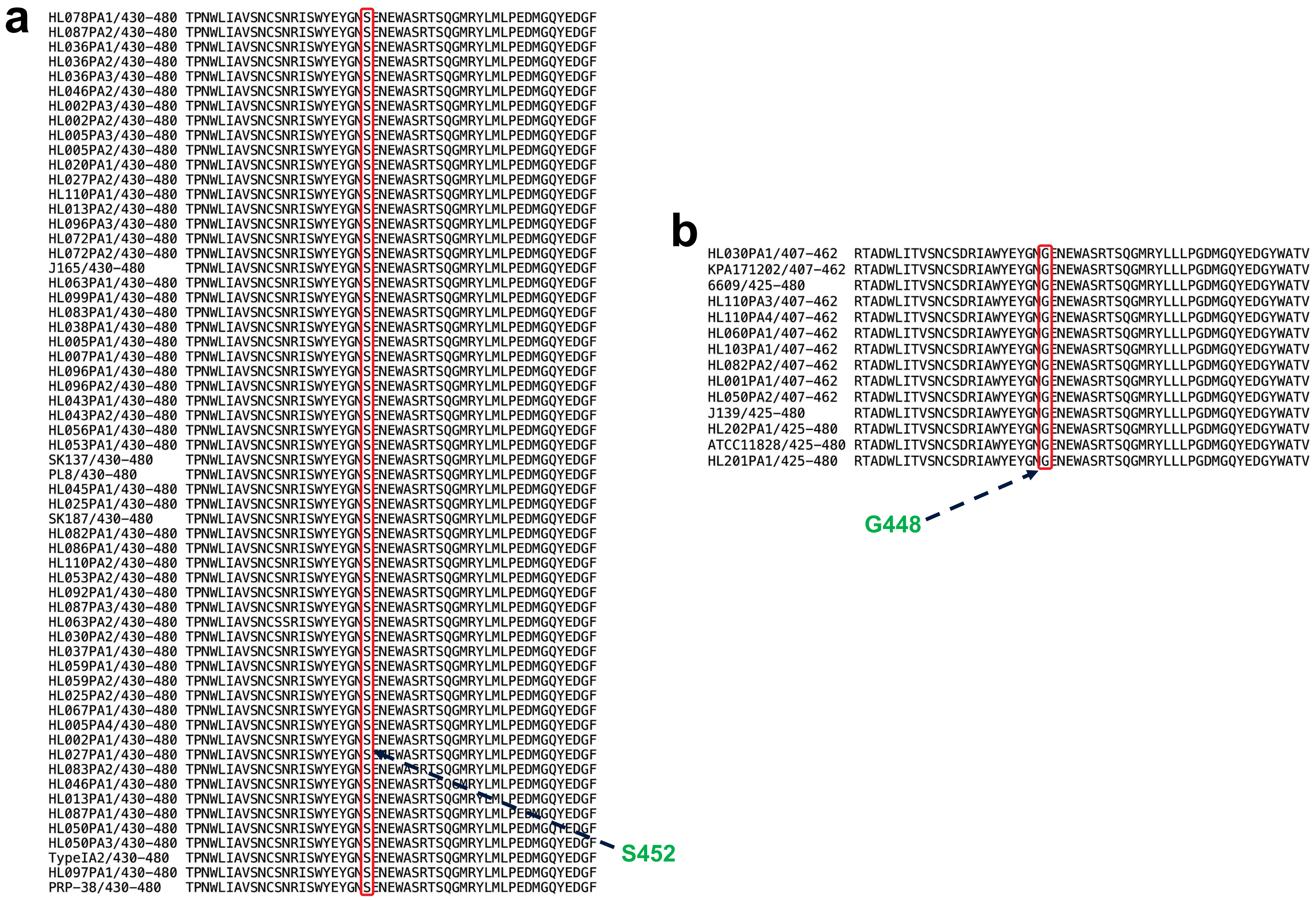

Supplementary Fig. 12: Sequence alignment of Hyl from *C. acnes* strains. Amino acid sequences of different *C. acnes* strains retrieved from the NCBI database. (**a**) Sequence alignment of HylA. The highly conserved residue Ser452 residue is highlighted in a red box (**b**) Sequence alignment of HylB. The highly conserved residue Gly448 (equivalent to the position of 452 in HylA) is highlighted in a red box. For simplicity, amino acid residues flanking G448 are shown.

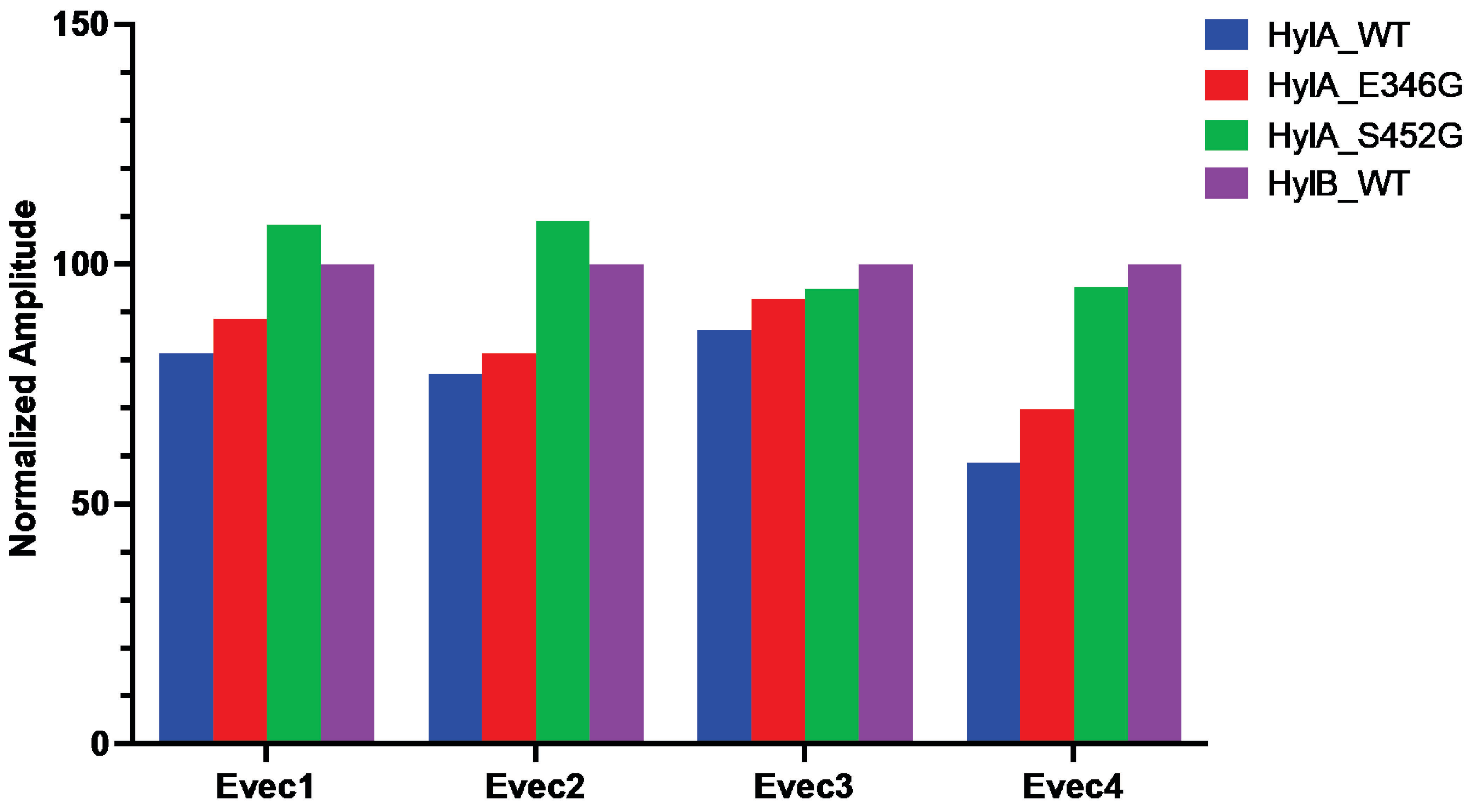

Supplementary Fig. 13: Domain motions in HylA-wt, HylB-wt and mutants of HylA. Normalized amplitudes of the four types of domain motions in each model are shown. The cleft opening/closing motion (Evec1), domain twisting motion (Evec2), substrate entry opening/closing motion (Evec3), and product-exit opening/closing motion(Evec4) were calculated from the molecular dynamics simulations by GROMACS version 2022.4.

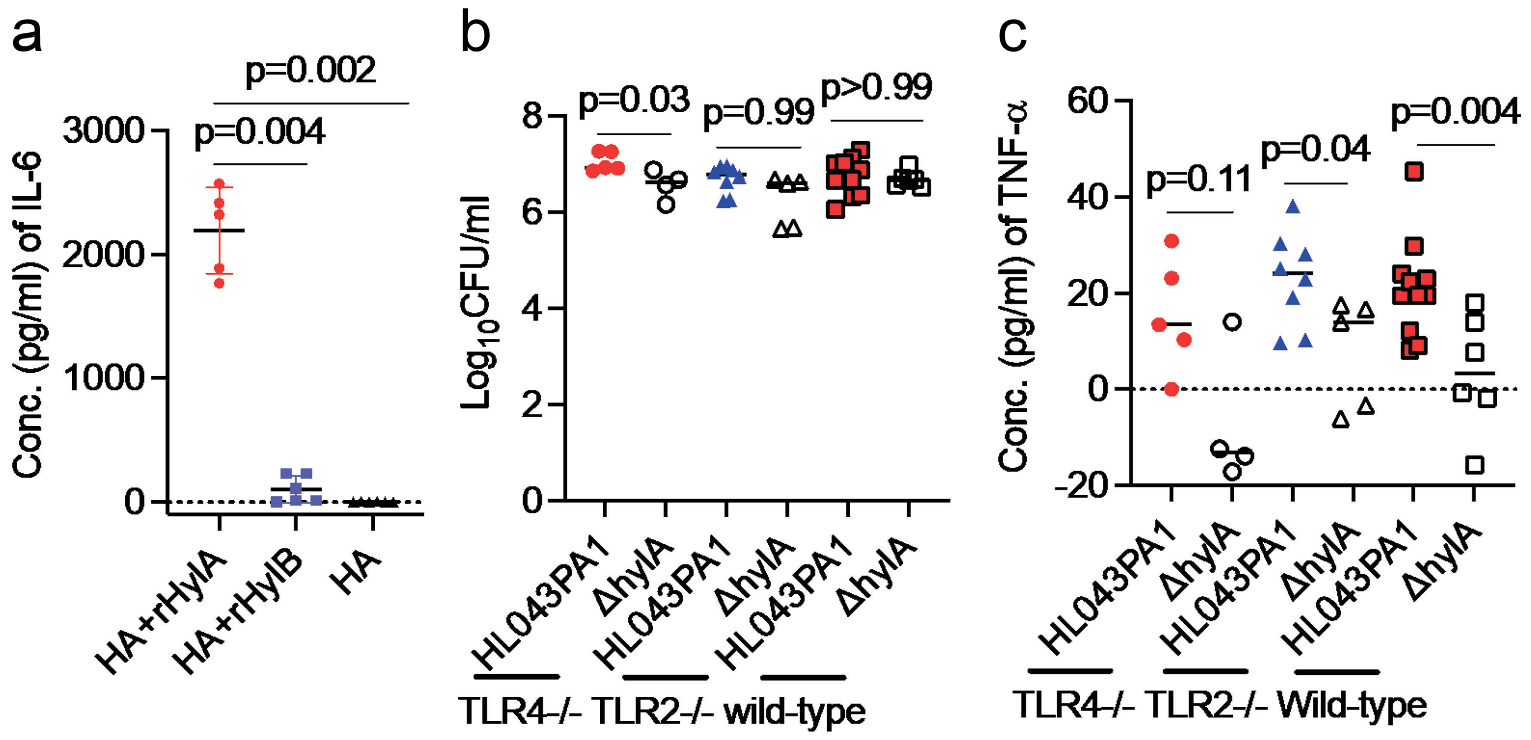

Supplementary Fig. 14: Proinflammatory properties of HylA- and HylB- cleaved HA fragments. a, C57Bl/6 mouse BMDMs were stimulated with either rHylA- or rHylB-digested HA. IL-6 cytokine in the culture supernatant was measured at 24 hr post-stimulation. **b, c**, C57Bl/6 WT, *TLR2*$^{-/-}$ or *TLR4*$^{-/-}$ mice were infected (2x10^{7}CFU) i.d. with HL043PA1 or isogenic *HylA*. Bacterial burden (**b**) and TNF-α (**c**) after 24 hr. Bar in **a** denotes mean ± SD and each data represents a single well (n=5 for HA + rHylA, and n=6 for HA + rHylB and HA). Data are representative of at least two experiments. Bars in **b, c** denote median, and each data point represents one mouse (n=5 *TLR4*$^{-/-}$, n=8 for *TLR2*$^{-/-}$ or n=11 for WT mice infected with HL043PA1, and n=4 for *TLR4*$^{-/-}$, n=5 for *TLR2*$^{-/-}$ or n=6 for WT mice infected with Δ*HylA)*. Data were analyzed by one-way ANOVA with Tukey`s post-hoc (**a**), or non-parametric two-tailed Mann-Whitney U test (**b, c**).

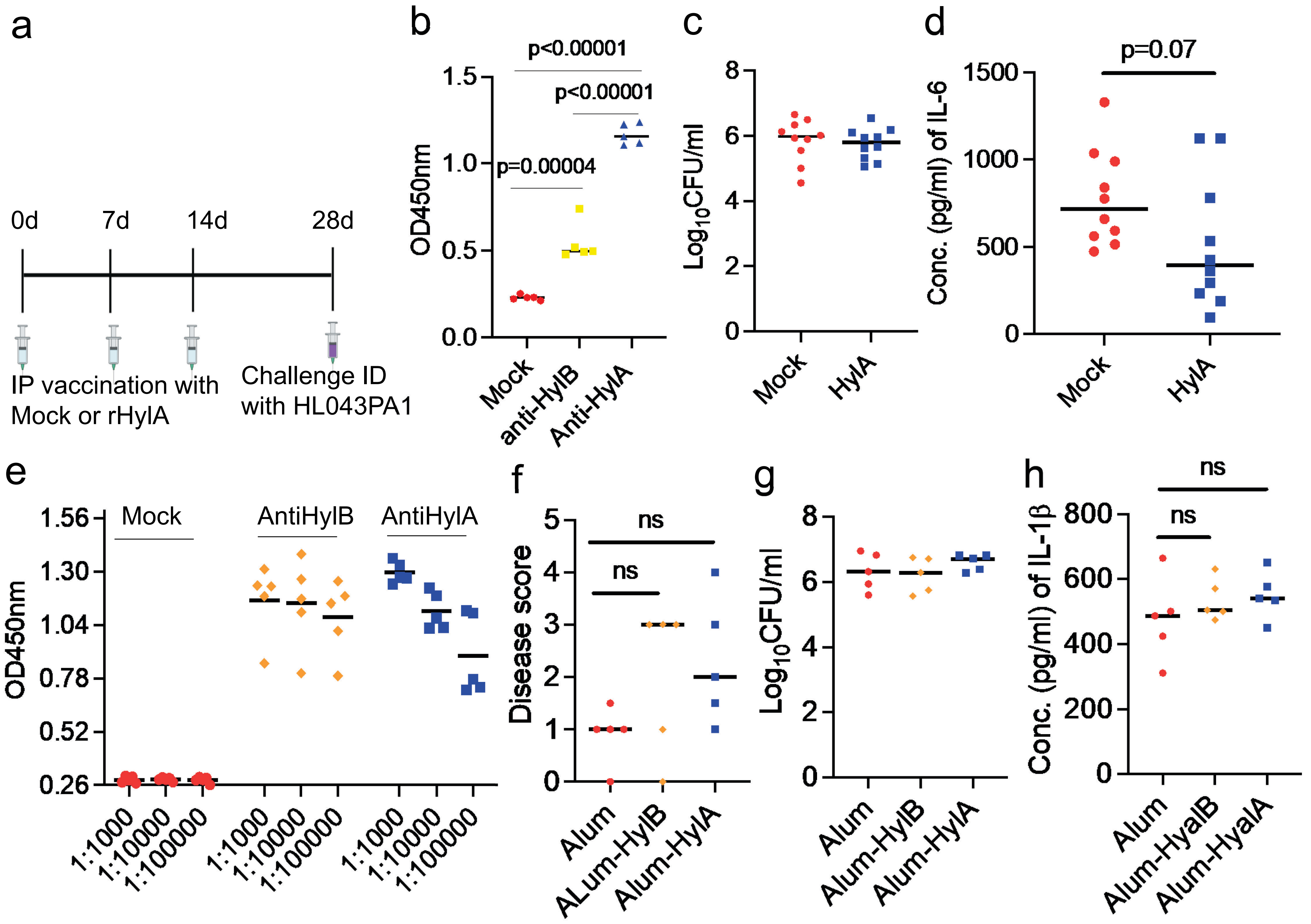

Supplementary Fig.15: rHylA vaccination protects mice against acne. a, Schematic of rHylA or HylB immunization followed by *C. acnes* challenge. **b,** Serum anti-HylA and anti-HylB antibody titers after the third rHylA immunization (n=5). **c-e,** CD1 mice (n=10) immunized with Alum (Mock) or Alum-rHylA (HylA) were challenged i.d. with HL043PA1. Bacterial burden (**c**) and IL-6 in skin lesions (**d**) at d2 post-challenge. **e**, titers of serum anti-HylA and anti-HylB from CD1 mice (n=5) immunized with rHylB. **f-h**, mice (n=5) were vaccinated with mock, rHylA, or rHylB and then challenged i.d. with HL110PA3. Disease score (**f**), bacterial burden (**g**) and IL-1β (**h**) at d 2 post-infection. Bars denote median. Data are from two independent experiments (**c,d**) or from one experiment (**b, e-h**) with each data point representing one mouse. The data in **b** were analysed by one-way ANOVA with Tukey`s post-hoc test, while the data in **c, d, f-h** were analyzed by non-parametric two-tailed Mann-Whitney U test.

a

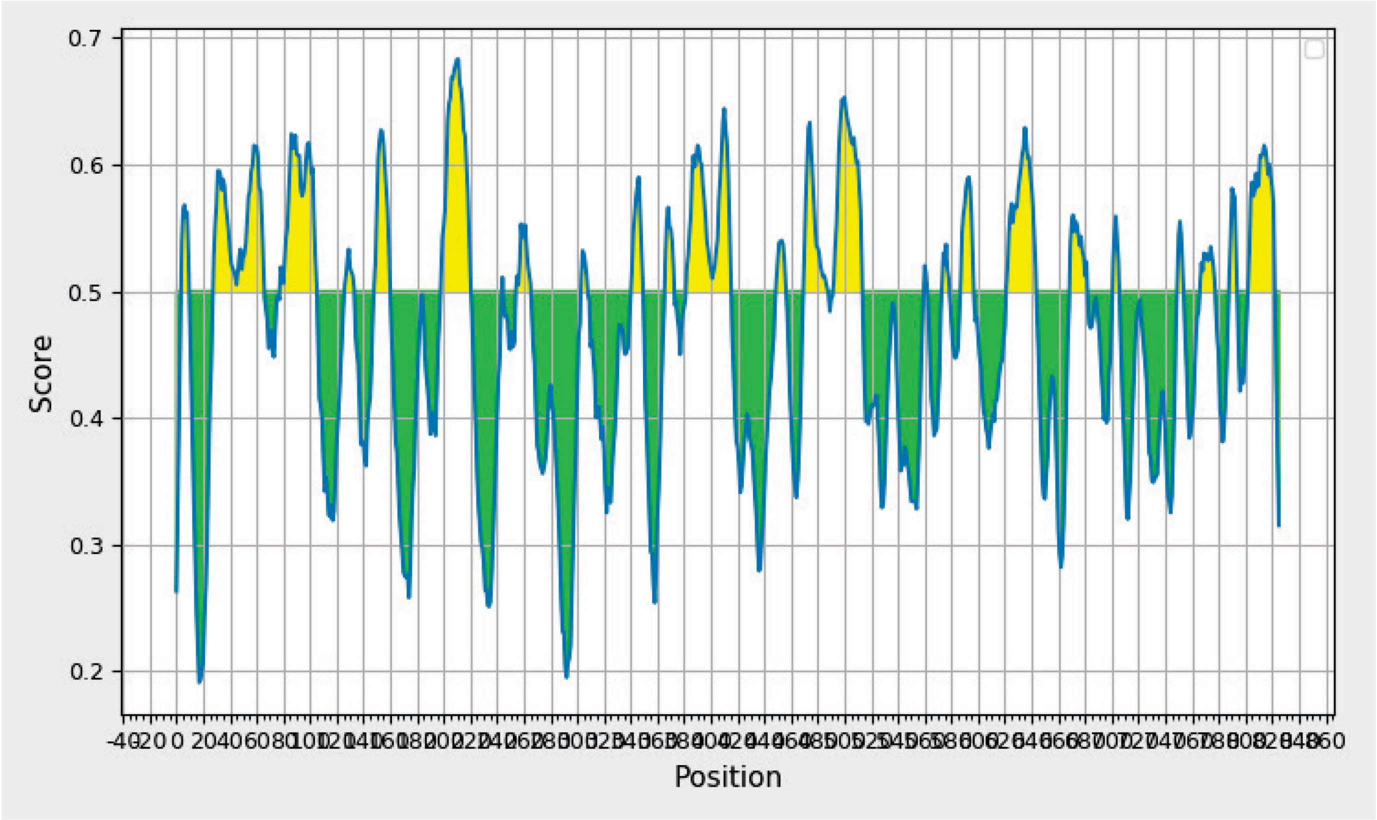

b

1	5	10	PYQVPS	6
2	29	66	PEMPDAFASPDPDIWSALCEKWTDIITGRNAAKTADPR	38
3	79	106	ATILTDLASSSSRTTVLLSANLQKEESS	28
4	127	134	SAYHAEPH	8
5	149	160	LRYHPSQDEYGN	12
6	199	221	VPDPWYQQPESVKPTAHPTQPVI	23
7	245	245	S	1
8	255	266	PDSWRTVAEGDG	12
9	304	308	RFDIT	5
10	341	350	ISRIDEPAAM	10
11	367	373	PAHRAEL	7
12	383	416	TRNTFDHLSEPASLRDIDLFDTAANVRPIPESST	34
13	450	457	GNSENEWA	8
14	471	488	EDMGQYEDGFWATVDYSA	18
15	493	514	TVDSTPLKRAVGTAWAERTPDN	22
16	561	563	TDA	3
17	574	579	KVGKTP	6
18	588	597	TITSKTSFDN	10
19	622	644	EKRKGSWIDVNPARTVKGFNEAI	23
20	670	682	RSQTMALAQRPVD	13
21	702	706	CLLTK	5
22	750	754	TQKRP	5
23	767	778	ENSSDRISVSRS	12
24	789	794	ADLGGQ	6
25	803	822	ALPKPTKPSLRASSYPLGLP	20

c

Query 6 (HyalA) YQVPSRRTFLSLSALSAIAIAASPEMPDAFASPDPDIWSALCEKWTDIITGRNAAKTADP 65
Subject 2 (HyalB) FGTPSRRTFLTASALSAMALAASPTVTDAIAAPGPDSWSALCERWIDIITGRRAARTSDP 61

Query 66 (HyalA) RARAIIAKTDKRVATILTDLASSSSRTTVLLSANLQKEESSFITTTARAISSIACAWATP 125
Subject 62 (HyalB) RARAIIAKTDRKVAEILTDLVSGSSRQTVLISADLRKEQSPFITKTARAIESMACAWATP 121

Query 726 (HyalA) TADAPAIIQTQAQGSRVEVIMSEPTQKRPSLTVAIEGVWTVENSSDRISVSRSDKTTTLR 785
Subject 722 (HyalB) AVNRPALVQTRESANQMEVVIVEPTQKRGSLTVTIEGSWKVKTADSHVDVSCENAAGTLH 781

Query 798 (HyalA) VTLSPALPKPTKPSLRASSYPLGLPHTSS 826

d

EMPDAFASPDPDIWGVATILTDLASSSSRTTVLLSANLQKEESSGENSSDRISVSRS
GALPKPTKPSLRASSYPLGLPGSITFLRDFWGNPLRYDTEYYLIPVASSSKDVQLKNI
TDYMYLTNAPSYTNGKLNIYYRRLYNGLKFIIKRYTPNNEIDSFVKSGDFIKLYVSYNN
NEHIVGYPKDGNAFNNLDRILRVGYNAPGIPLYKKMEAVKLRDLKTYSVQLKLYDDK
NASLGLVGTHNGQIGNDPNRDILIASNWYFNHLKDKILGCDWYFVPTDEGWTND

Supplementary Fig. 16: Design of HylA multi-epitope (mEHylA) vaccine. a,b, Linear B cell epitopes in HylA protein were predicted using Bepipred Linear Epitope Prediction 2.0 (IEDB analysis resources, http://tools.iedb.org/bcell/). The chart shows immunogenic peptides with scores above 0.5 (**a**) and table shows list of predicted peptides (**b**). **c**, Alignment of 4 selected predicted peptides (in red) shows no sequence homology with HylB. **d**, the 4 predicted epitopes (in red) were physically linked to the C-terminus of tetanus toxoid protein (in blue). A linker amino acid glycine (G) was placed between each peptide and the C-terminus of tetanus.

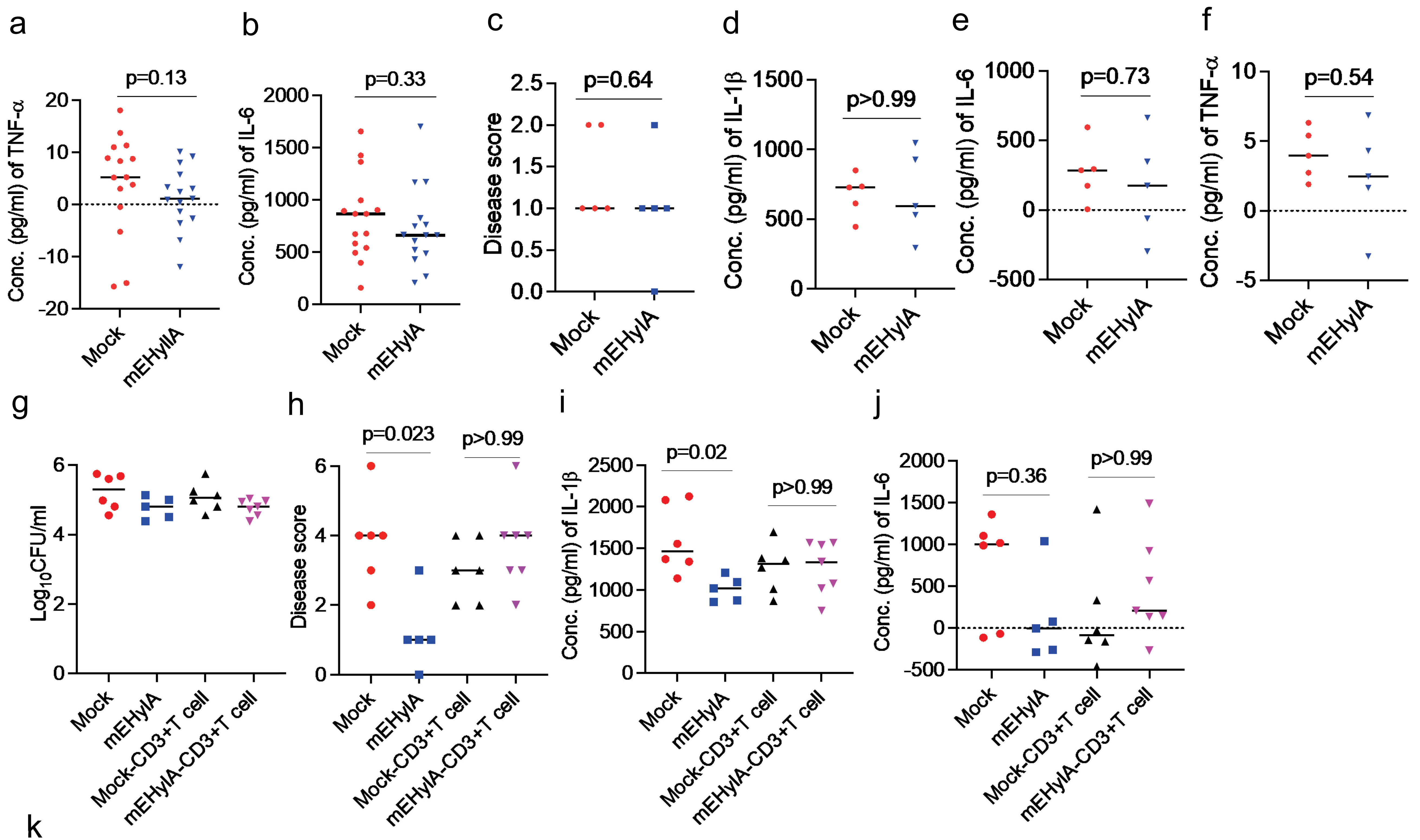

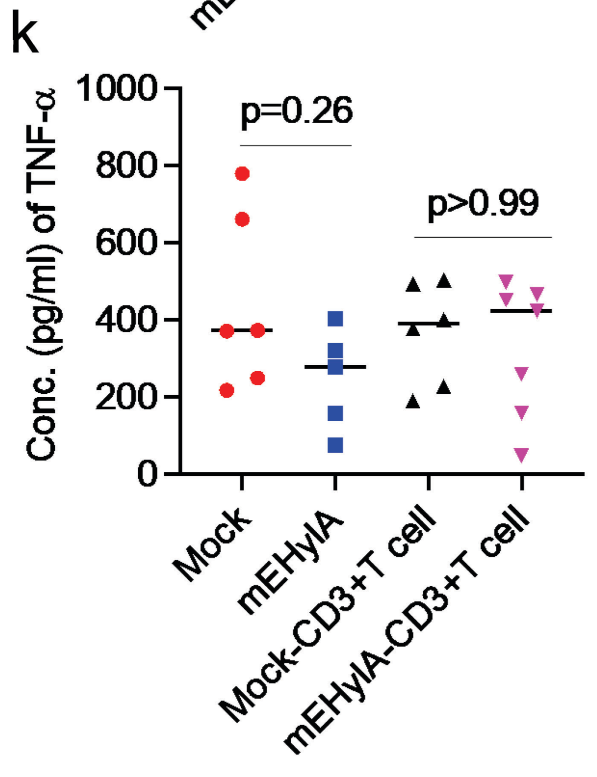

Supplementary Fig. 17: Effect of mEHylA vaccination on disease induced by HL043PA1 or HL110PA3 infection. a-b, mice (n=15) were vaccinated, as in Supplementary Fig. 15, with either mock (Alum-TT) or Alum plus mEHylA (mEHylA) and challenged 14d post-last vaccination with HL043PA1. TNF-α (**a**) and IL-6 (**b**) at d2 post-infection. **c-f**, mice (n=5) were vaccinated, as above with either mock (Alum-TT) or Alum plus mEHylA (mEHylA) and challenged 14d post-last vaccination with HL110PA3. Disease score (**c**), IL-1β (**d**), IL-6 (**e**), and TNF-α (**f**) at d2 post-infection. **g-k**, CD1 mice (n=5) were vaccinated as above with either mock (Alum-TT) or Alum plus mEHylA (mEHylA), and then 10d post-last vaccination total CD3+T cells were isolated from spleen and adoptive transferred into naïve recipient mice, followed by HL043PA1 challenge 20h later. CFUs (**g**), disease score (**h**) and cytokines (**i-k**) were measured on d2 post-infection. Bars denote median. Data are from three independent (**a-b**) or from one experiment (**c-k**), with each data point representing one mouse. The p values in **a-f** were calculated by non-parametric two-tailed Mann-Whitney U test and by one-way ANOVA for **g-k**.

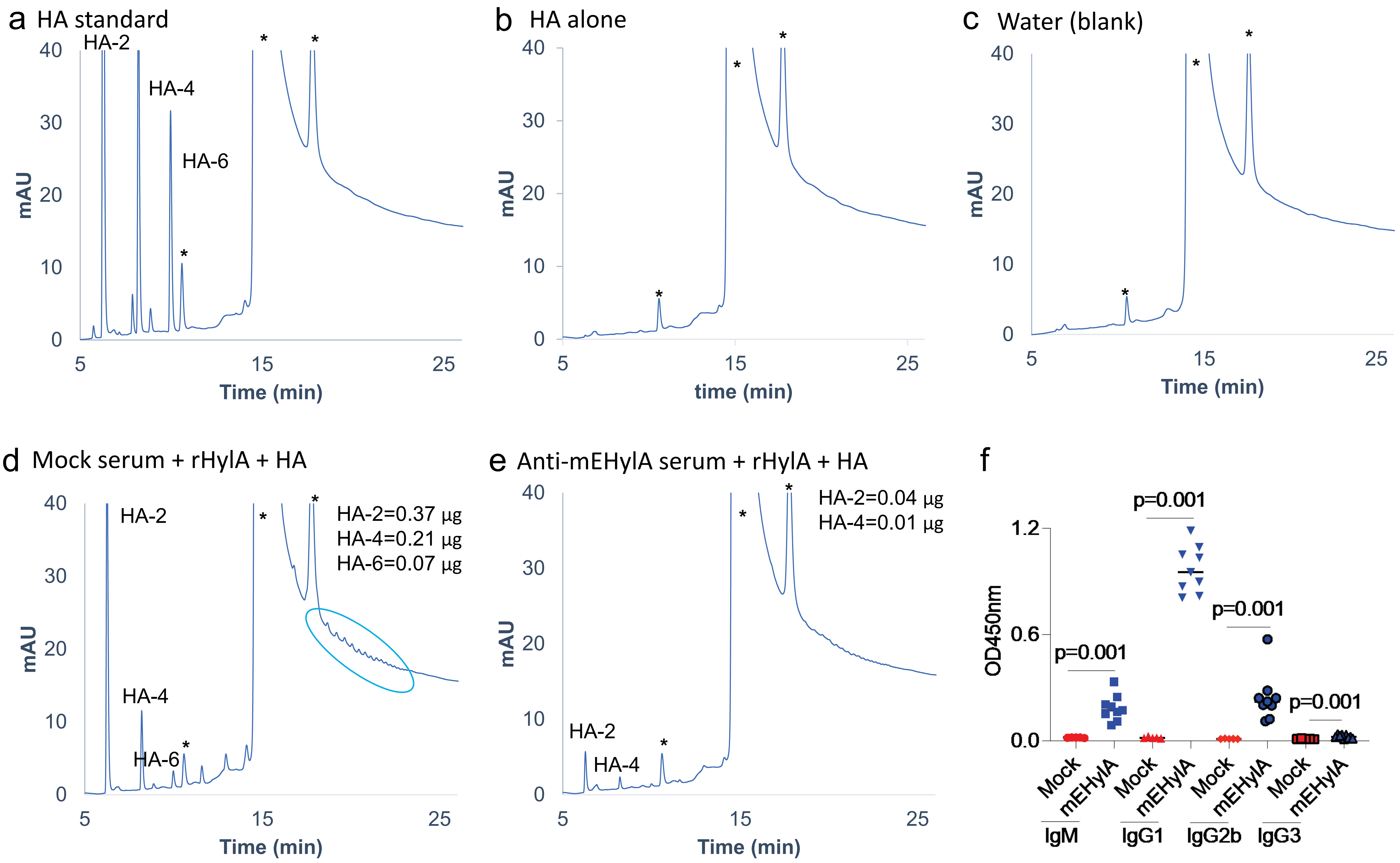

Supplementary Fig. 18 HylA enzyme neutralization assay. a-e, HPLC profile of HMW-HA (2 mg/ml) after 20 hr coincubation with rHylA (0.3 µg) and serum (10 µl): HA standard (**a**), HA alone (**b**), water ran as blank (**c**), Mock serum (Alum-TT) + rHylA + HA (**d**), and anti-mEHylA serum + rHylA + HA (**e**). Asterisk (*) in **a-e** represents non-specific peaks, present in water control as well. Pooled serum (n=5) was used for the assay and performed in duplicate for mock serum and in triplicate for anti-mEHylA serum. **f**) Antibody isotype titers (serum diluted 1:100,000) in sera isolated from mice vaccinated with either mock (Alum-TT) or Alum plus mEHylA (mEHylA) at d14 post-last vaccination. Statistical analysis in **f** was performed by one-way Anova with Tukey`s post-hoc test. Green circle represents larger oligomers.

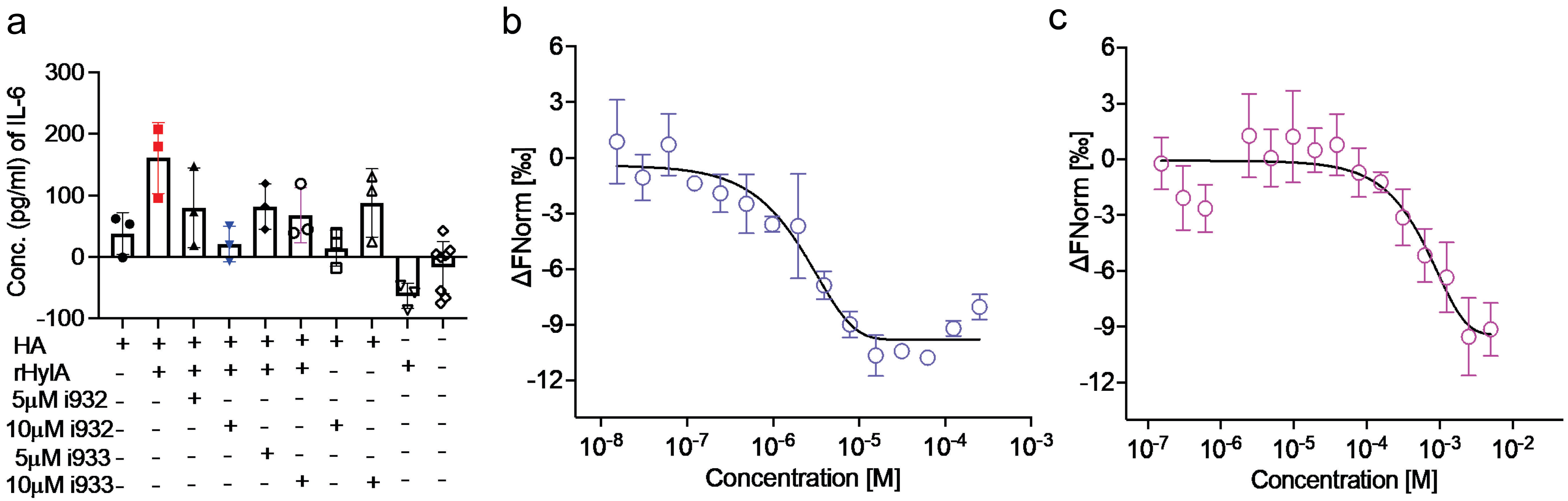

Supplementary Fig. 19: HylA-Inhibitors ameliorate inflammation in human keratinocytes. a, HMW HA was digested with rHylA in the presence or absence of inhibitors i932 or i933, then applied to HaCaT cells for 24 hr. Shown are IL-6 from culture supernatant. Data presented as mean ± SD. Each data point in **a**, is a technical replicate. Experiment was performed 3 times and results are shown of one independent experiment. The data were analyzed by one-way ANOVA with Tukey `s post-hoc test. **b, c,** microscale thermophoresis (MST) analysis of HylA binding to the peptide inhibitors i933 **(b)** and i93 **(c).** The MST dose response curves were obtained by titrating the peptide against the fluorescent labeled HylA and the data were fit to the Kd model. The graphs in **b, c** show the normalized fluorescence of the peptide binding to the protein. The MST data is represented as mean +/- SD of triplicates. The data is collected in 3 individual experiments, which are essentially technical replicates, due to the technical limitations of the MST method.

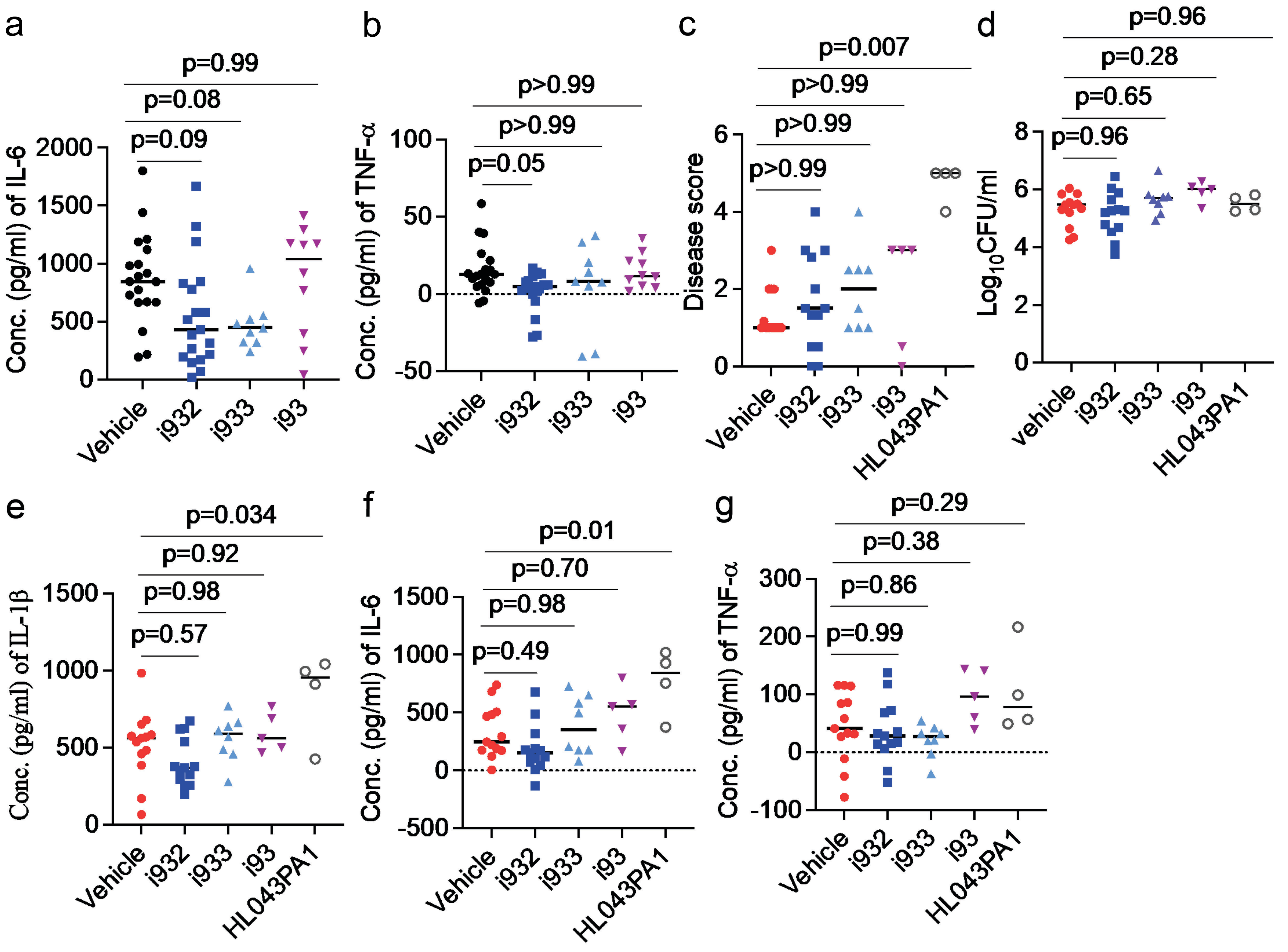

Supplementary Fig. 20: Selective neutralization of HylA improves acne lesions and mitigates inflammation. a, b, 10 µg Inhibitors (i932, i933 or i93) and HL043PA1 strain (2 x 10^7CFU/mouse) were co-injected into CD1 mice (n=19 for vehicle and i932, n=9 for i933 and n=10 for i93). IL-6 (**a**) and TNF-α (**b**) in the skin lesion at d1 post infection. **c-g,** 10 µg inhibitors (i932, i933 or i93) and HL110PA3 strain (2 x 10^7CFU/mouse) were co-injected i.d. into CD1 mice (n=4 for HL043PA1, n=5 for i93, n=8 for i933 and n=13 for vehicle and i932). Disease score (**c**), bacterial burden (**d**) and cytokines (**e-g**) at d1 post-infection**.** HL043PA1 alone (n=4) served as a control in **c-g.** Bars denote median. The data are from two independent experiments with each data point representing one mouse. Data were analyzed by one-way ANOVA with Tukey`s post-hoc test.

Supplementary Table 1: Phylotype of *C. acnes*, and the presence of *Hyl* (*A* or *B*) gene, association with acne or healthy skin, and the ribotype represented [6, 11, 12]

Phylotype Clades	Phylotypes by McDowell [12]	Phylotypes by Lomholt [11]	*Hyl*	Health/acne association	Major Ribotype represented
**IA-1**	IA1	I-1a	*A*	Acne	1
**IA-2**	IA1	I-1a	*A*	Acne	4/5
**IB-1**	IA1	I-1b	*A*	Acne	8
**IC**	IC	na	*A*	Acne	5
**II**	II	II	*B*	Healthy skin	2/6

Supplementary Table 2: Crystallographic data collection, processing, and refinement statistics (Molecular replacement)

Parameter	HylB WT	HylB Y281F	HylA Y285F
PDB code	8FNX	8G0O	8FYG
**Data collection**			
Space group	P 41 21 2	P 1	P 1
Unit cell constants			
a, b, c (Å)	103.68,103.68, 172.94	52.98, 53.01, 161.02	51.96, 59.42, 125.99
α, β, γ (°)	90.00, 90.00, 90.00	91.95, 98.03, 114.56	90.61, 95.74, 90.08
Resolution range (Å)	19.73 - 2.1 (2.21-2.1)[#]	19.72 - 2.1 (2.21-2.1)	19.8 - 2.05 (2.1-2.05)
Data completeness (%)	99.70 (97.8)	92.6 (87.7)	93.9 (90.59)
Total reflections	784293 (102767)	161839 (21056)	200582 (16051)
Unique reflections	55667 (7843)	85175 (11885)	88266 (6191)
Multiplicity	14.1 (13.1)	1.9 (1.8)	2.2 (1.9)
Mean I/sigma(I)	14.50 (4.3)	5.4 (1.7)	7.33 (1.99)
Wilson B-factor (Å^2)	18.04	15.3	23.30
R-merge	0.172 (0.672)	0.12 (0.548)	0.097 (0.497)
R-meas	0.178 (0.699)	0.17 (0.775)	0.128 (0.662)
CC1/2	0.997 (0.892)	0.981 (0.853)	0.991 (0.623)
CC*	0.999 (0.967)	0.995 (0.894)	0.998 (0.893)
**Refinement**			
Reflections used in refinement	55667 (7843)	85104 (8160)	88252 (5832)
Reflections used for R-free	2000 (283)	2061 (235)	2006 (138)
R-work	0.1564 (0.2034)	0.2525 (0.339)	0.2252 (0.319)
R-free	0.1930 (0.2527)	0.2685 (0.355)	0.2579 (0.35)
CC(work)	0.969 (0.914)	0.893 (0.674)	0.931 (0.713)
CC(free)	0.955 (0.820)	0.875 (0.595)	0.904 (0.681)
**No. of non-hydrogen atoms**			
Total	6842	12210	12845
Macromolecules	5830	11540	11485
Ligands	52	0	28
Solvent	960	670	1332
Protein residues	762	1519	1522
Rotamer outliers (%)	0.49	1.58	0.50
Clashscore	2.57	6.40	6.36
Average B-factor (Å^2)			
Overall	20.96	21.64	26.90
Macromolecules	19.29	21.69	26.37
Ligands	36.49	N/A	27.62
Solvent	30.23	20.80	32.04
**R.m.s. deviations**			
Bond lengths (Å)	0.007	0.003	0.002
Bond angles (°)	0.83	0.60	0.55

Values in the parentheses are for highest-resolution shell.

Supplementary Table 3: Primers used for cloning rHylA, rHylB proteins (top 4) and constructing *ΔhylA* and *ΔhylB C. acnes* strains (bottom 10)

Primer name	Sequence (5`-3`)
hylA forward	TACTTCCAATCCAATGCA GATATTTGGTCGGCACTGTGC
hylA reverse	TTATCCACTTCCAATGTTATTAGGGCAGCGCAGGGGACAGTG
hylB forward	TACTTCCAATCCAATGCAGACAGCTGGTCGGCGCTGTGC
hylB reverse	TTATCCACTTCCAATGTTATTAAGTTACCTGGCGTGCCAGCGT
hylA F1 up	GAAGTGAAGCAACAAAGCAAGTGA
hylA R1 up	CGCGGTACCTAATCGCGCTCCTAGAGCAAG
hylA F2 down	CGCGGTACCGCAATAAAAGTAGGCCCCTGG
hylA R2 down	GCGATGACATTAAGGTGTCCTCCC
hylB F1 up	CGTCAGGGCCAATGAACAGGCCTC
hylB R1 up	CGCGGTACCGGGTCACGCTCCTCGGGAGAGGGT
hylB F2 down	CGCGGTACCTGTGCCGCCTCACCTAACTAG
HylB R1 down	GCAGTGGGCTTCTCGGTGTAGATG
erm Forward	CCGGGTACCAGAAGGTCGAACTCG
erm reverse	GCAGGTACCAGCCCGACCCGAGCA

Supplementary Table 4: HPLC gradient conditions

Time(min)	%A	%B
2	100	0
25	75	25
27	40	60
32	100	0
45	100	0

Supplementary table 5: Primers used for cloning the HylA and HylB mutants

Mutation	Forward primer (5'→3')	Reverse primer (5'→3')
Mutations in hyla gene		
HylA S116E	CCACAACCGCCCGTGCCATTGAATCCATCGCGTGCGCCTGGG	CCCAGGCGCACGCGATGGATTCAATGGCACGGGCGGTTGTGG
HylA S284G	AATACGTCGCCGTAGCCGCCGGTGTAAGGGAC	GTCCCTTACACCGGCGGCTACGGCGACGTATT
HylA Y285F	CGTCCCTTACACCGGCTCCTTCGGCGACGTATTGCTGAGCGGC	GCCGCTCAGCAATACGTCGCCGAAGGAGCCGGTGTAAGGGACG
HylA D345N	GCCGCTCCATCTCGCGCATCAATGAACCCGCAGCCATGCACGG	CCGTGCATGGCTGCGGGTTCATTGATGCGCGAGATGGAGCGGC
HylA E346G	GCCGCTCCATCTCGCGCATCGATGGCCCCGCAGCCATGCACGGCATGTCC	GGACATGCCGTGCATGGCTGCGGGGCCATCGATGCGCGAGATGGAGCGGC
HylA R397V	CCTGAGTGAACCTGCCTCGCTGGTGGATATCGATCTCTTCGACACC	GGTGTCGAAGAGATCGATATCCACCAGCGAGGCAGGTTCACTCAGG
HylA N442D	CGTCTCTAACTGCTCGGATCGCATCTCGTGGTACG	CGTACCACGAGATGCGATCCGAGCAGTTAGAGACG
HylA S452G	GTGGTACGAGTATGGCAACGGCGAAAATGAGTGGGCGTCAA	TTGACGCCCACTCATTTTCGCCGTTGCCATACTCGTACCAC
Mutations in hylb gene		
HylB R62D	CGCAGAGCCGCCCGGACCTCTGACCCAGACGCCCGAGCGATCATCGCCAAGACCGA	GGTCGGTCTTGGCGATGATCGCTCGGGCGTCTGGGTCAGAGGTCCGGGCGGCTCTGC
HylB R109A	GCAGTCGCCCTTCATCACCAAGACAGCCGCTGCCATCGAGTCGATGGCCTGCGCCTG	CCCAGGCGCAGGCCATCGACTCGATGGCAGCGGCTGTCTTGGTGATGAAGGGCGACT
HylB E112S	CCAAGACAGCCCGAGCCATCTCGTCGATGGCCTGCGCCTGGG	CCCAGGCGCAGGCCATCGACGAGATGGCTCGGGCTGTCTTGG
HylB Y281F	CCACATCCCCTACACCGGCGGCTTCGGCGACGTCCTGTTCAGCGG	CCGCTGAACAGGACGTCGCCGAAGCCGCCGGTGTAGGGGATGTGG
HylB N341D	CGTGCGCGGCCGATCCATCTCGCGCATCGACGGGTCTGCCGCCATGCACGGCATCTC	CGAGATGCCGTGCATGGCGGCAGACCCGTCGATGCGCGAGATGGATCGGCCGCGCA
HylB G342E	GCCGATCCATCTCGCGCATCAACGAATCTGCCGCCATGCACGGCATCTCG	CGAGATGCCGTGCATGGCGGCAGATTCGTTGATGCGCGAGATGGATCGGC
HylB M357L	CGATCGCCCGTGCCATGCTCCTCATGGCTGATGCCCTGCCGACACACC	GGTGTGTCGGCAGGGCATCAGCCATGAGGAGCATGGCACGGGCGATCG
HylB L386A	GGATGGCTCGAAACACCTTCGATCACGCTTCCGAGCCGTCCACCCTTGTCGACATCT	GGAGATGTCGACAAGGGTGGACGGCTCGGAAGCGTGATCGAAGGTGTTTCGAGCCAT
HylB S390P	CGAAACACCTTCGATCACCTGTCCGAGCCGCCCACCCTTGTCGACATCTCCCTGTTC	CGAACAGGGAGATGTCGACAAGGGTGGGCGGCTCGGACAGGTGATCGAAGGTGTTTC
HylB V393R	CCTGTCCGAGCCGTCCACCCTTCGCGACATCTCCCTGTTCGACG	CGTCGAACAGGGAGATGTCGCGAAGGGTGGACGGCTCGGACAGG
HylB F398A	GCCGTCCACCCTTGTCGACATCTCCCTGGCTGACGCCGCCGCCAAGGCGCGCCCCG	GGACGGGGCGCGCCTTGGCGGCGGCGTCAGCCAGGGAGATGTCGACAAGGGTGGAC
HylB R405A	CCCTGTTCGACGCCGCCGCCAAGGCGGCGCCCGTCCCGGAGTCGTCGACGCCG	CGGCGTCGACGACTCCGGGACGGGCGCCGCCTTGGCGGCGGCGTCGAACAGGG
