# Supplementary material for: Functional divergence of a bacterial enzyme promotes healthy or acneic skin
Source: Nat Commun. 2023 Dec 5;14:8061. doi: 10.1038/s41467-023-43833-8 (PMC10697930; doi:10.1038/s41467-023-43833-8)
Supplement: Supplementary file 3 — Reporting Summary [file 41467_2023_43833_MOESM3_ESM.pdf]

nature portfolio

Corresponding author(s): George Liu
Ramachandran Murali

Last updated by author(s): Nov. 6, 2023

Reporting Summary

Nature Portfolio wishes to improve the reproducibility of the work that we publish. This form provides structure for consistency and transparency in reporting. For further information on Nature Portfolio policies, see our Editorial Policies and the Editorial Policy Checklist.

Statistics

For all statistical analyses, confirm that the following items are present in the figure legend, table legend, main text, or Methods section.

n/a	Confirmed	
☐	☒	The exact sample size (*n*) for each experimental group/condition, given as a discrete number and unit of measurement
☐	☒	A statement on whether measurements were taken from distinct samples or whether the same sample was measured repeatedly
☐	☒	The statistical test(s) used AND whether they are one- or two-sided<br>*Only common tests should be described solely by name; describe more complex techniques in the Methods section.*
☒	☐	A description of all covariates tested
☐	☒	A description of any assumptions or corrections, such as tests of normality and adjustment for multiple comparisons
☐	☒	A full description of the statistical parameters including central tendency (e.g. means) or other basic estimates (e.g. regression coefficient) AND variation (e.g. standard deviation) or associated estimates of uncertainty (e.g. confidence intervals)
☐	☒	For null hypothesis testing, the test statistic (e.g. F, t, r) with confidence intervals, effect sizes, degrees of freedom and P value noted<br>*Give P values as exact values whenever suitable.*
☒	☐	For Bayesian analysis, information on the choice of priors and Markov chain Monte Carlo settings
☒	☐	For hierarchical and complex designs, identification of the appropriate level for tests and full reporting of outcomes
☒	☐	Estimates of effect sizes (e.g. Cohen's d, Pearson's r), indicating how they were calculated

Our web collection on statistics for biologists contains articles on many of the points above.

Software and code

Policy information about availability of computer code

Data collection	HylA and HylB FASTA amino acid sequences were obtained from NCBI or the RCSB protein.
Data analysis	XDS version Jun 1, 2017 BUILT=20170923 for processing the X-ray diffraction data.<br>SCALA version 3.3.21 for scaling.<br>XSCALE version Jun 1, 2017 BUILT=20170923 for scaling.<br>PHASER version 2.5.6 for molecular replacement.<br>COOT version 0.9.6 for model building.<br>PHENIX version 1.20.1_4487 for refinement.<br>PyMOL version 2.4 (The PyMOL Molecular Graphics System, Version 2.4 Schrödinger, LLC.) for the preparation of structural figures.<br>SBGrid Consortium platform[www.sbgrid.org] for running all the crystallographic and structure visualization & analysis tools.<br>Dali Server[http://ekhidna2.biocenter.helsinki.fi/dali/] for pairwise structural comparisons.<br>GraphPad Prism version 8<br>GROMACS version 2022.4 for analyzing the molecular dynamics simulations data.<br>Schrodinger software package version 2021-1 (Schrodinger, inc. San Diego, CA) for analyzing the structure-based virtual screening data.<br>MAESTRO version 12.8 for preparing the protein for the structure-based virtual screening.<br>Clustal Omega server[https://www.ebi.ac.uk/Tools/msa/clustalo/] for multiple sequence alignment of the amino acid sequences

For manuscripts utilizing custom algorithms or software that are central to the research but not yet described in published literature, software must be made available to editors and reviewers. We strongly encourage code deposition in a community repository (e.g. GitHub). See the Nature Portfolio guidelines for submitting code & software for further information.

Data

Policy information about availability of data

All manuscripts must include a data availability statement. This statement should provide the following information, where applicable:

- Accession codes, unique identifiers, or web links for publicly available datasets
- A description of any restrictions on data availability
- For clinical datasets or third party data, please ensure that the statement adheres to our policy

Any data generated or analyzed during this study, associated protocols, materials within the manuscript and public databases (PDB database) are included in the article and also available from the corresponding authors upon request. The authors have filed a patent application for HylA and HylB bacterial mutants, recombinant HylA and HylB proteins, inhibitors targeting HylA (i93, i932 and i933), and a vaccine construct (mEHylA), and these materials may be available under restrictive conditions. Source data are provided with this paper. Structural data from crystallographic studies are available from RCSB Protein Data Bank; the PDB codes for the crystal structures HylA (8FYG[https://www.rcsb.org/structure/unreleased/8FYG]) and HylB (8FNX[https://www.rcsb.org/structure/unreleased/8FNX], 8G0O[https://www.rcsb.org/structure/unreleased/8G0O]).

Research involving human participants, their data, or biological material

Policy information about studies with human participants or human data. See also policy information about sex, gender (identity/presentation), and sexual orientation and race, ethnicity and racism.

Reporting on sex and gender	Not relevant to this study
Reporting on race, ethnicity, or other socially relevant groupings	Not relevant to this study
Population characteristics	Not relevant to this study
Recruitment	Not relevant to this study
Ethics oversight	Not relevant to this study

Note that full information on the approval of the study protocol must also be provided in the manuscript.

Field-specific reporting

Please select the one below that is the best fit for your research. If you are not sure, read the appropriate sections before making your selection.

☒ Life sciences ☐ Behavioural & social sciences ☐ Ecological, evolutionary & environmental sciences

For a reference copy of the document with all sections, see nature.com/documents/nr-reporting-summary-flat.pdf

Life sciences study design

All studies must disclose on these points even when the disclosure is negative.

Sample size	Sample size was selected based on the published literature. To apply statistical analysis, minimum number of animals allocated to a group was 3. Please see the reference.
Data exclusions	No data was excluded from the analysis.
Replication	The number of experimental replicates is indicated in the respective figure legends. All in vitro experiments were repeated at least more than once. Most of the animal experiments were performed with minimum sample size 3 and maximum 19. The details are provided in the respective figure legends.
Randomization	Age-matched animals (n=5 per cage) were randomly assigned to experimental groups and then accordingly treated as follows: infection with P. acnes alone, infection with P. acnes + inhibitors, infection with P. acnes + vehicle. In case of vaccination, animals were treated as Mock (Alum or Alum + tetanus toxoid protein) or vaccine (Alum + HylA, Alum + mEHylA).
Blinding	The experiments were not blinded. Two independent researchers confirmed the disease phenotype in animals obtained with HylA and HylB mutants. Vaccination with HylA was confirmed by two independent researches. HPLC analysis of HA degradation product by HylA and HylB was confirmed by two independent researchers. All animals were treated under identical conditions with proper controls and phenotype was confirmed by a replicate experiment.

Reporting for specific materials, systems and methods

We require information from authors about some types of materials, experimental systems and methods used in many studies. Here, indicate whether each material, system or method listed is relevant to your study. If you are not sure if a list item applies to your research, read the appropriate section before selecting a response.

Materials & experimental systems

n/a	Involved in the study
☐	☒ Antibodies
☐	☒ Eukaryotic cell lines
☒	☐ Palaeontology and archaeology
☐	☒ Animals and other organisms
☒	☐ Clinical data
☒	☐ Dual use research of concern
☒	☐ Plants

Methods

n/a	Involved in the study
☒	☐ ChIP-seq
☒	☐ Flow cytometry
☒	☐ MRI-based neuroimaging

Antibodies

Antibodies used	Goat Anti-Mouse IgG, Human ads-HRP (Cat. No.: 1030-05),<br>Goat Anti-Mouse IgG1, Human ads-HRP (Cat. No.: 1070-05),<br>Goat Anti-Mouse IgG2b, Human ads-HRP (Cat. No.: 1090-05),<br>Goat Anti-Mouse IgG3, Human ads-HRP (Cat. No.: 1100-05)<br>Goat Anti-Mouse IgM, Human ads-HRP (Cat. No.: 1020-05)<br>Plus serum antibodies against HylA generated from experimental vaccines
Validation	Verified by the supplier (SouthernBiotech) for reactivity to mouse.

Eukaryotic cell lines

Policy information about cell lines and Sex and Gender in Research

Cell line source(s)	HaCaT cell line was purchased from ATCC
Authentication	Verified by ATCC at the time of distribution via series of morphology, karyotyping and PCR approaches.
Mycoplasma contamination	Cell lines were negative to mycoplasma (tested and verified by ATCC)
Commonly misidentified lines<br>(See ICLAC register)	No commonly misidentified cell lines were used in this study.

Animals and other research organisms

Policy information about studies involving animals; ARRIVE guidelines recommended for reporting animal research, and Sex and Gender in Research

Laboratory animals	Six weeks-old C57BL/6, TLR2−/− (Strain #:004650), and TLR4-/- (Strain #:004650) mice were purchased from Jackson Laboratories. TLR2-/- and TLR4-/- mice were bred in specific-pathogen free facilities. All mice (Mus musculus) were kept in filter-top cages with access to food pellet and water under controlled ambient temperatures (20-22oC), relative humidity (30-70%) and 12 h light/12 h dark cycle. The animal experiments were performed at approximately 8 weeks of age unless otherwise specified. For preparation of BMDMs, 12 weeks-old mice were used.
Wild animals	No wild animals were used in this study
Reporting on sex	only female mice were used in this study.
Field-collected samples	No field collected samples were used in this study.
Ethics oversight	All animal studies were approved under the guidelines of the University of California San Diego (UCSD) Institutional Animal Care and Use Committee (IRB animal protocol approved number S18200). The mice were housed in an animal facility at UCSD with a standard of care as per federal, state, local, and NIH guidelines.

Note that full information on the approval of the study protocol must also be provided in the manuscript.

Plants

Seed stocks	Not applicable
Novel plant genotypes	Not applicable
Authentication	Not applicable
